# Disruption of USP9X in macrophages promotes foam cell formation and atherosclerosis

**DOI:** 10.1172/JCI154217

**Published:** 2022-05-16

**Authors:** Biqing Wang, Xuening Tang, Liu Yao, Yuxin Wang, Zhipeng Chen, Mengqi Li, Naishi Wu, Dawei Wu, Xiangchen Dai, Hongfeng Jiang, Ding Ai

**Affiliations:** 1 The State Key Laboratory of Experimental Hematology, National Clinical Research Center for Blood Diseases, Key Laboratory of Immune Microenvironment and Disease (Ministry of Education), Province and Ministry Co-sponsored Collaborative Innovation Center for Medical Epigenetics, Tianjin Institute of Cardiology, The Second Hospital of Tianjin Medical University and; 2 Department of Physiology and Pathophysiology, Tianjin Medical University, Tianjin, China.; 3 Key Laboratory of Remodeling-Related Cardiovascular Diseases (Ministry of Education), Beijing Collaborative Innovation Center for Cardiovascular Disorders, Beijing Institute of Heart, Lung and Blood Vessel Diseases, Beijing Anzhen Hospital, Capital Medical University, Beijing, China.; 4 Department of Cardiovascular Surgery, Tianjin Medical University General Hospital, Tianjin, China.

**Keywords:** Cardiology, Immunology, Atherosclerosis, Macrophages, Ubiquitin-proteosome system

## Abstract

Subendothelial macrophage internalization of modified lipids and foam cell formation are hallmarks of atherosclerosis. Deubiquitinating enzymes (DUBs) are involved in various cellular activities; however, their role in foam cell formation is not fully understood. Here, using a loss-of-function lipid accumulation screening, we identified ubiquitin-specific peptidase 9 X-linked (USP9X) as a factor that suppressed lipid uptake in macrophages. We found that USP9X expression in lesional macrophages was reduced during atherosclerosis development in both humans and rodents. Atherosclerotic lesions from macrophage USP9X-deficient mice showed increased macrophage infiltration, lipid deposition, and necrotic core content than control apolipoprotein E–KO (*Apoe^–/–^*) mice. Additionally, loss-of-function USP9X exacerbated lipid uptake, foam cell formation, and inflammatory responses in macrophages. Mechanistically, the class A1 scavenger receptor (SR-A1) was identified as a USP9X substrate that removed the K63 polyubiquitin chain at the K27 site. Genetic or pharmacological inhibition of USP9X increased SR-A1 cell surface internalization after binding of oxidized LDL (ox-LDL). The K27R mutation of SR-A1 dramatically attenuated basal and USP9X knockdown–induced ox-LDL uptake. Moreover, blocking binding of USP9X to SR-A1 with a cell-penetrating peptide exacerbated foam cell formation and atherosclerosis. In this study, we identified macrophage USP9X as a beneficial regulator of atherosclerosis and revealed the specific mechanisms for the development of potential therapeutic strategies for atherosclerosis.

## Graphical abstract

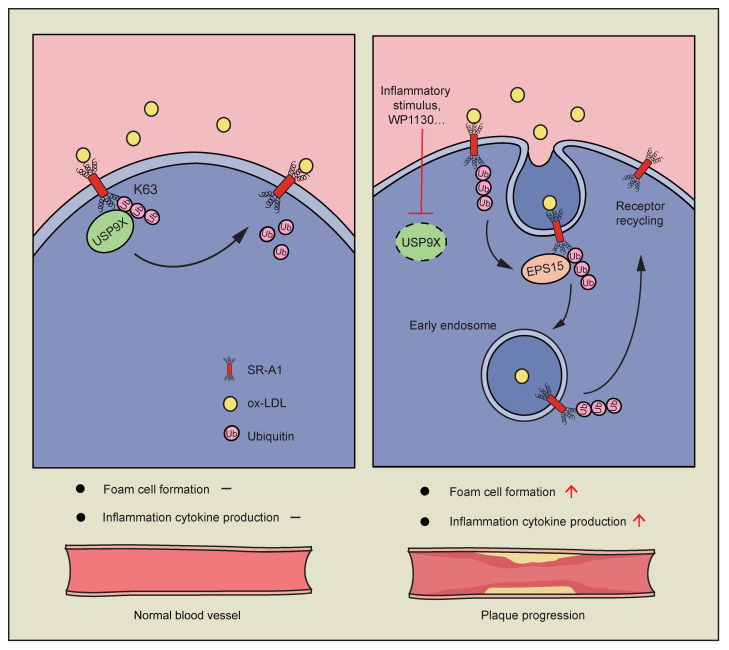

## Introduction

Atherosclerosis is a chronic inflammatory disease characterized by excessive cholesterol deposition in the walls of large and medium arteries. It is the leading cause of various cardiovascular diseases and the main contributor to acute cardiovascular events, such as myocardial infarction and stroke (1). During the development of atherosclerosis, circulating monocytes migrate to the subintimal layer of the artery and differentiate into macrophages (2). These cells engulf large amounts of oxidized LDL (ox-LDL) via scavenger receptors on their surface, resulting in their transformation into lipid-rich foam cells. Foam cells produce ROS and induce inflammatory responses to recruit more macrophages into the vascular intima, resulting in fat streaks in the blood vessel wall and accelerated progression of atherosclerosis (3, 4). Thus, a better understanding of the mechanism underlying foam cell formation may lead to the discovery of new therapeutic targets for atherosclerosis. Macrophages are an important source of foam cells in atherosclerosis (5, 6). Disorders of lipid metabolism in macrophages, such as excessive uptake of ox-LDL and impaired cholesterol outflow, cause macrophage foaming (7). Macrophages ingest extracellularly modified LDL through receptor-mediated phagocytosis and pinocytosis. In vitro data showed that CD36 and the class A1 scavenger receptor (SR-A1) account for 75% to 90% of the total internalized ox-LDL in macrophages (8). The inhibition of CD36 and SR-A1 expression in macrophages significantly reduced foam cell formation (9, 10). SR-A1 is a trimeric integral membrane glycoprotein, which upon binding with ox-LDL to form a ligand-receptor complex is endocytosed to form vesicles and transferred to the endosome. As the ligand is released, SR-A1 recycles back to the cell surface. The intracellular domain of SR-A1 contains 55 amino acid residues that play a role in membrane transport, recycling, and internalization (11). Mutations in the cytoplasmic domain of SR-A1 significantly reduce SR-A1–mediated cellular internalization of modified LDL and cell adhesion (12, 13). Although SR-A1 is one of the main scavenger receptors for ox-LDL uptake, the mechanism of ligand-induced SR-A1 internalization and the corresponding downstream signals remain unclear.

Hermann et al. found that ubiquitin immunoreactivity was enhanced in unstable plaque areas in patients with acute coronary syndrome (14). Moreover, ubiquitin-proteasome system hyperactivity is related to the enhancement of plaque inflammatory responses and plaque vulnerability (15). These findings suggest that the ubiquitin-proteasome system is involved in the development of atherosclerosis. Ubiquitination plays an important role in the endocytosis of various surface receptors, which is a critical step in foam cell formation. Deubiquitinating enzymes (DUBs) are proteases that cleave ubiquitin molecules from ubiquitin-connected substrates. Using experimental and bioinformatics methods, nearly 100 DUBs have been identified in humans, but most are not well characterized. DUBs are divided into 5 main families, 4 of which are cysteine proteases consisting of the ubiquitin C-terminal hydrolase (UCH), ubiquitin-specific protease (USP/UBP), otubain domain ubiquitin-binding protein (OTU), and the Josephin domain– or Machado-Joseph domain–containing protease (MJD). The fifth member is a protease containing the Jab1/Pab1/MPN domain (JAMM), which belongs to the metalloprotease family (16). However, the role of DUBs in foam cell formation and atherosclerosis is still unclear.

Ubiquitin-specific peptidase 9 X-linked (USP9X) has been reported to target several cytosolic proteins and regulate multiple cellular activities, such as protein endocytosis, immune responses, and neurogenesis. Studies have shown that USP9X mediates epsin-specific deubiquitination to regulate vesicle trafficking (17). USP9X also controls the internalization of activated EGFR by interacting with Itch and Eps15 (18, 19). In addition, USP9X has been implicated in several neurodevelopmental and neurodegenerative disorders and malignancies. CircRNA hsa_circ_0090231 (circ-*USP9X*) is derived from the *USP9X* gene, which was elevated in ox-LDL–treated endothelial cells and promoted endothelial cell injury via the miR-599/CLIC4 axis (20). However, the role of USP9X protein in foam cell formation and atherogenesis has rarely been reported.

In this study, we used a functional genomics approach to screen for DUBs that reduce lipid accumulation and lipid uptake in macrophages. We found that USP9X regulated foam cell formation and was negatively associated with atherosclerosis in rodents and humans. Mechanistically, we showed that USP9X inhibition enhanced K63-linked polyubiquitination of SR-A1, and this posttranslational modification promoted SR-A1 internalization, foam cell formation, and inflammatory responses in macrophages. Thus, our findings provide potentially new insights into the atheroprotective effect of macrophage USP9X.

## Results

### USP9X is identified as a foam cell formation regulator by functional genomics screening.

**Figure 1:**
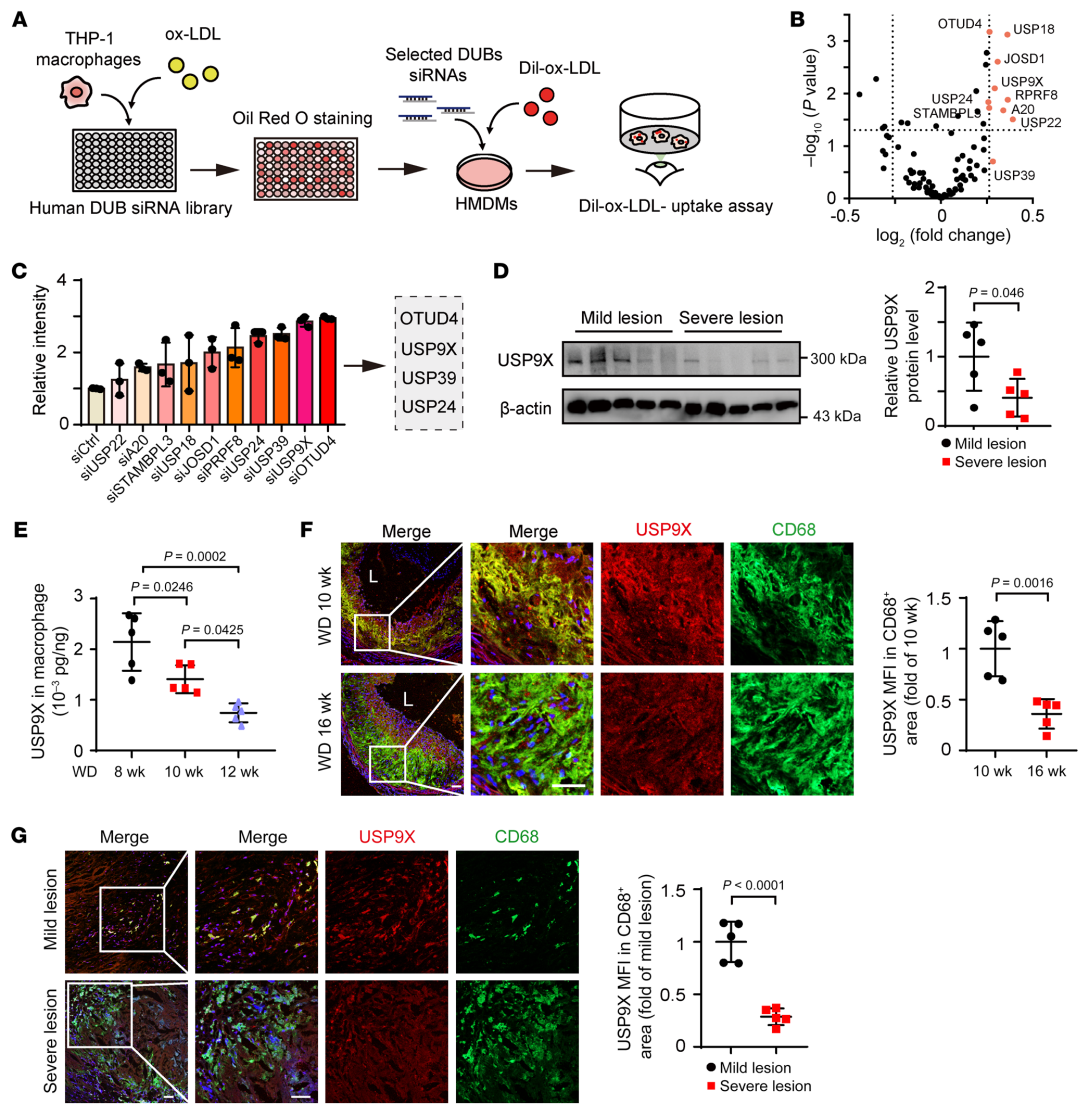
USP9X is identified as a suppressor of foam cell formation and negatively correlated with atherosclerosis. (**A**) Schematic diagram of the process used to screen for DUBs regulating transformation of macrophages into foam cells and lipid uptake. (**B**) Oil Red O staining of macrophages transfected with DUB-specific siRNAs. Volcano plot showing fold-change in intensity of staining. Labels indicate significantly upregulated genes. (**C**) Human monocyte–derived macrophages (HMDMs) were transfected with DUB-specific siRNAs for 48 hours followed by treatment with Dil-ox-LDL (30 μg/mL) for 4 hours. Cellular fluorescence intensity was measured (*n =* 3). (**D**) Western blot analysis of the levels of USP9X proteins in mild and severe atherosclerotic lesions in human carotid arteries. Paired 2-tailed Student’s *t* test (*n =* 5). (**E**) ELISA of USP9X protein levels in macrophages of the aorta from *Apoe^–/–^* mice fed a Western diet (WD) for 8, 10, and 12 weeks. One-way ANOVA with Bonferroni’s multiple-comparison post hoc test (*n =* 5). (**F**) *Apoe^–/–^* mice were fed a WD for 10 and 16 weeks. Immunofluorescence analysis of USP9X and CD68 expression in aortic root sections (left). Quantification of the fluorescence intensity of USP9X in CD68^+^ areas (right). Scale bar: 50 μm. Unpaired 2-tailed Student’s *t* test (*n =* 5). (**G**) Immunofluorescence analysis of USP9X and CD68 expression in severe and mild atherosclerotic lesions in human carotid arteries (left). Data represent the fluorescence intensity of USP9X in CD68^+^ areas (right). Scale bar: 50 μm. Paired 2-tailed Student’s *t* test (*n =* 5).

To identify foam cell formation–associated DUBs, we performed a functional genomics screen of a panel of 98 DUBs from known DUB families (5 UCHs, 51 USPs, 3 MJDs, 9 OTUs, 6 JAMMs, and others) using a human DUB-specific siRNA library in the THP-1–derived macrophages (Figure 1A and Supplemental Figure 1A; supplemental material available online with this article; https://doi.org/10.1172/JCI154217DS1). Oil Red O staining indicated that some DUBs were involved in the macrophage lipid accumulation process (Figure 1B and Supplemental Figure 1B). Of these, loss of function in 10 candidates was found to increase intracellular lipid accumulation by more than 1.2-fold compared with that of the control group; these were selected for subsequent validation. To determine the role of these DUBs in lipid uptake, we knocked down these 10 genes in human monocyte–derived macrophages (HMDMs) and tracked lipid uptake using Dil-labeled ox-LDL. Among the 10 candidates, *OTUD4-*, *USP9X-*, *USP39-*, and *USP24*-deficient cells showed more than 2-fold increases in Dil-ox-LDL uptake (Figure 1C).

To reveal the clinical importance of our findings, we measured the expression of these 4 DUBs in human mild and severe atherosclerotic lesions. As shown in Figure 1D, USP9X protein levels in human severe atherosclerotic plaques were reduced compared with those in mild atherosclerotic plaques, whereas the levels of OTUD4, USP39, and USP24 proteins were comparable between the 2 groups (Supplemental Figure 1C). Next, we examined the expression of USP9X in lesional macrophages from *Apoe^–/–^* mice fed a Western diet (WD) for different time periods. We found that *Apoe^–/–^* mice fed a WD for 10 weeks had a 34% reduction in USP9X protein levels compared with those fed the same diet for 8 weeks, with a further decrease to a 65% reduction after 12 weeks (Figure 1E). However, there were no significant differences in *Usp9x* mRNA expression between the 3 groups (Supplemental Figure 1D). USP9X expression was further analyzed in atherosclerotic lesions by immunofluorescence staining. In WD-fed mice, USP9X expression was reduced predominantly in CD68^+^ cells of atherosclerotic lesion areas in a time-dependent manner (Figure 1F). There was no significant change in USP9X expression in the smooth muscle cell marker αSMA^+^ areas with extended WD feeding time (Supplemental Figure 1, E and F). In addition, USP9X expression in human CD68^+^ cells of severe plaques was lower than that in mild plaques (Figure 1G). These findings indicate that macrophage USP9X was reduced via a positive feedback loop to increase foam cell formation during the development of atherosclerosis.

Atherosclerosis is a chronic inflammatory disease of the arteries, and inflammation promotes foam cell formation and contributes to atherosclerosis (21–24). To investigate the role of USP9X in the inflammatory response of macrophages, we treated mouse bone marrow–derived macrophages (BMDMs) with 3 well-established inflammation inducers: IFN-γ, TNF-α, and LPS. Our results showed that TNF-α and LPS treatment reduced the expression of USP9X, suggesting that USP9X might play a role in the inflammatory response of macrophages (Supplemental Figure 1G).

### Macrophage USP9X deficiency promotes atherosclerosis.

**Figure 2:**
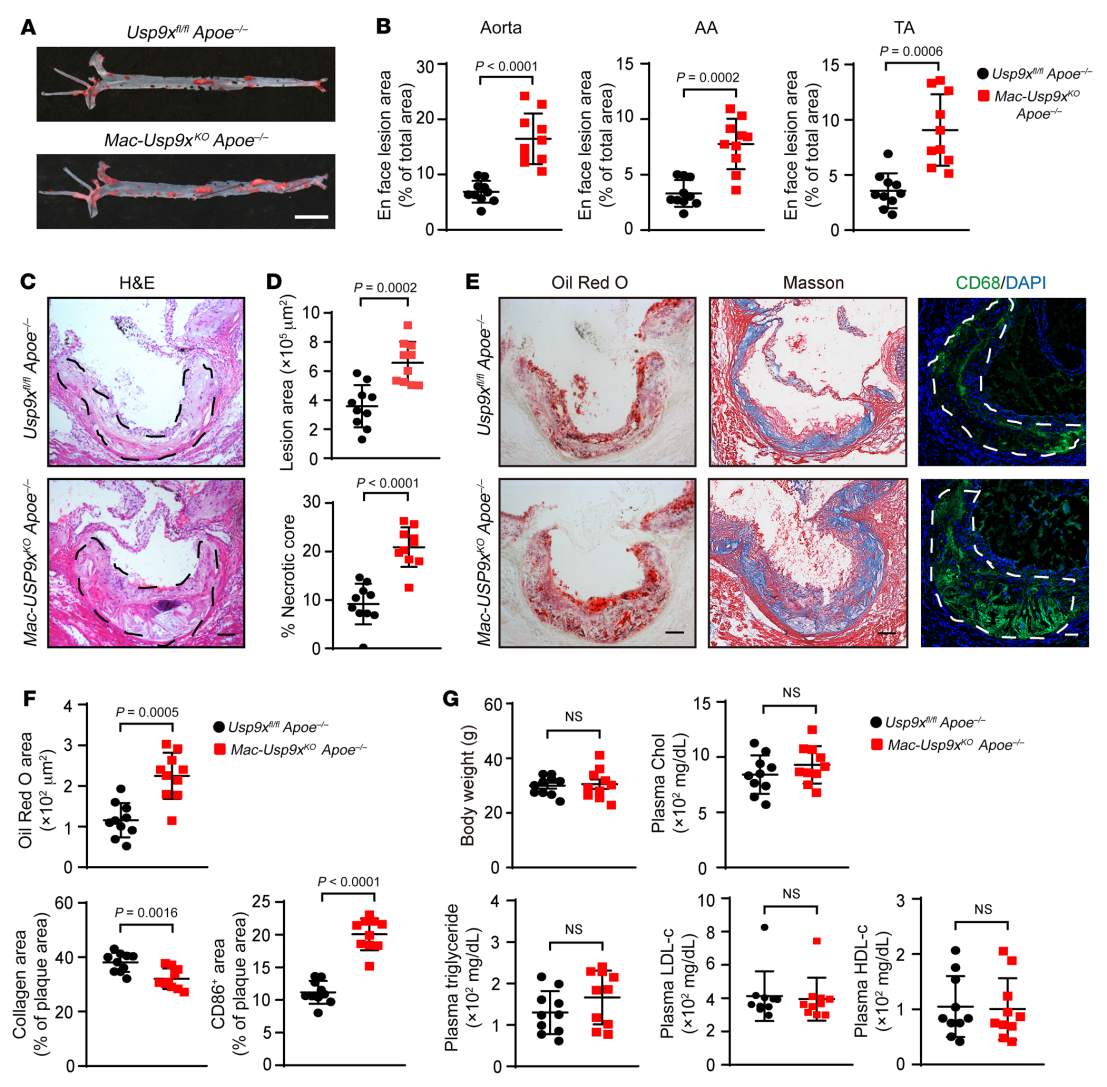
USP9X deficiency accelerates the development of atherosclerosis. (**A**) Oil Red O staining of aortas from female *Apoe^–/–^* and *Mac Usp9x^KO^ Apoe^–/–^* mice fed a Western diet (WD) for 16 weeks (*n =* 10). Scale bar: 5 mm. (**B**) Data represent the percentage of plaque area/total vessel area. AA, aortic arch; TA, thoracic aorta. Unpaired 2-tailed Student’s *t* test (*n =* 10). (**C**) H&E staining of representative aortic root sections. Black dashed lines demarcate atherosclerotic plaques (*n =* 10). Scale bar: 100 μm. (**D**) Quantification of lesion area and percentage of necrotic core. Unpaired 2-tailed Student’s *t* test (*n =* 10). (**E**) Oil Red O (left) and Masson’s trichrome (middle) staining of aortic root sections. Scale bar: 100 μm. Macrophages identified by anti-CD68 antibody staining (right). The white dashed line indicates plaques. Scale bar: 50 μm. Unpaired 2-tailed Student’s *t* test (*n =* 10). (**F**) Quantification of Oil Red O, collagen, and CD68^+^ areas in plaques. Unpaired 2-tailed Student’s *t* test (*n =* 10). (**G**) Body weight. Mann-Whitney *U* test (*n =* 10). Plasma levels of triglycerides (TGs), cholesterol (CHO), LDL-C, and HDL-C. Unpaired 2-tailed Student’s *t* test (*n =* 10). NS, not significant.

To explore the role of macrophage USP9X in the formation of atherosclerotic plaques, we crossed *Usp9x^fl/fl^ Apoe^–/–^* mice with *LysM-Cre* mice to generate *Apoe^–/–^* mice with *Usp9x*-deficient myeloid cells; for simplicity, we refer to these mice as *Mac Usp9x^KO^ Apoe^–/–^* mice while recognizing that the KO is not completely macrophage specific. After 16 weeks of WD, Oil Red O staining of aortas revealed that the atherosclerotic lesion area in the whole aorta of *Mac Usp9x^KO^ Apoe^–/–^* mice was significantly greater than that of *Usp9x^fl/fl^ Apoe^–/–^*mice (Figure 2, A and B). Furthermore, the atherosclerotic lesion area in the aortic root of *Mac Usp9x^KO^ Apoe^–/–^*mice was increased by 83%, and the necrotic core area was significantly increased compared with that of *Usp9x^fl/fl^* mice (Figure 2, C and D). We then conducted a more detailed analysis of aortic root components. Compared with *Usp9x^fl/fl^* mice, morphological analyses of the cross-sectional lesions showed a 15% decrease in collagen content and 94% and 71% increases in lipid accumulation and macrophage contents, respectively, in *Mac Usp9x^KO^ Apoe^–/–^* mice (Figure 2, E and F). Body weight and levels of plasma triglycerides (TGs), total cholesterol, LDL-C, and HDL-C were comparable between the two groups (Figure 2G). A similar trend was observed in male mice (Supplemental Figure 2, A–G). These results indicate that the lack of USP9X in macrophages accelerated the process of atherosclerosis.

### Macrophage USP9X regulates lipid uptake and inflammatory responses.

Because Cre-recombinase driven by the *LysM* promoter is expressed in both macrophages and neutrophils (25), we first explored the role of USP9X in neutrophils. Compared with macrophages, neutrophils expressed much lower levels of USP9X (Supplemental Figure 3A). Furthermore, *Apoe^–/–^* mice fed a WD for 8, 10, and 12 weeks exhibited comparable levels of USP9X protein expression in neutrophils (Supplemental Figure 3B). In addition, in vitro neutrophil function assays revealed similar migration, ROS production, and phagocytosis abilities of WT and *Usp9x*-deficient neutrophils (Supplemental Figure 3, C–F). Taken together, the lack of influence of *Usp9x* on key neutrophil functions suggested that macrophages might play a major role in the proatherosclerotic phenotype in *Mac* *Usp9x^ko^ ApoE^–/–^* mice.

**Figure 3:**
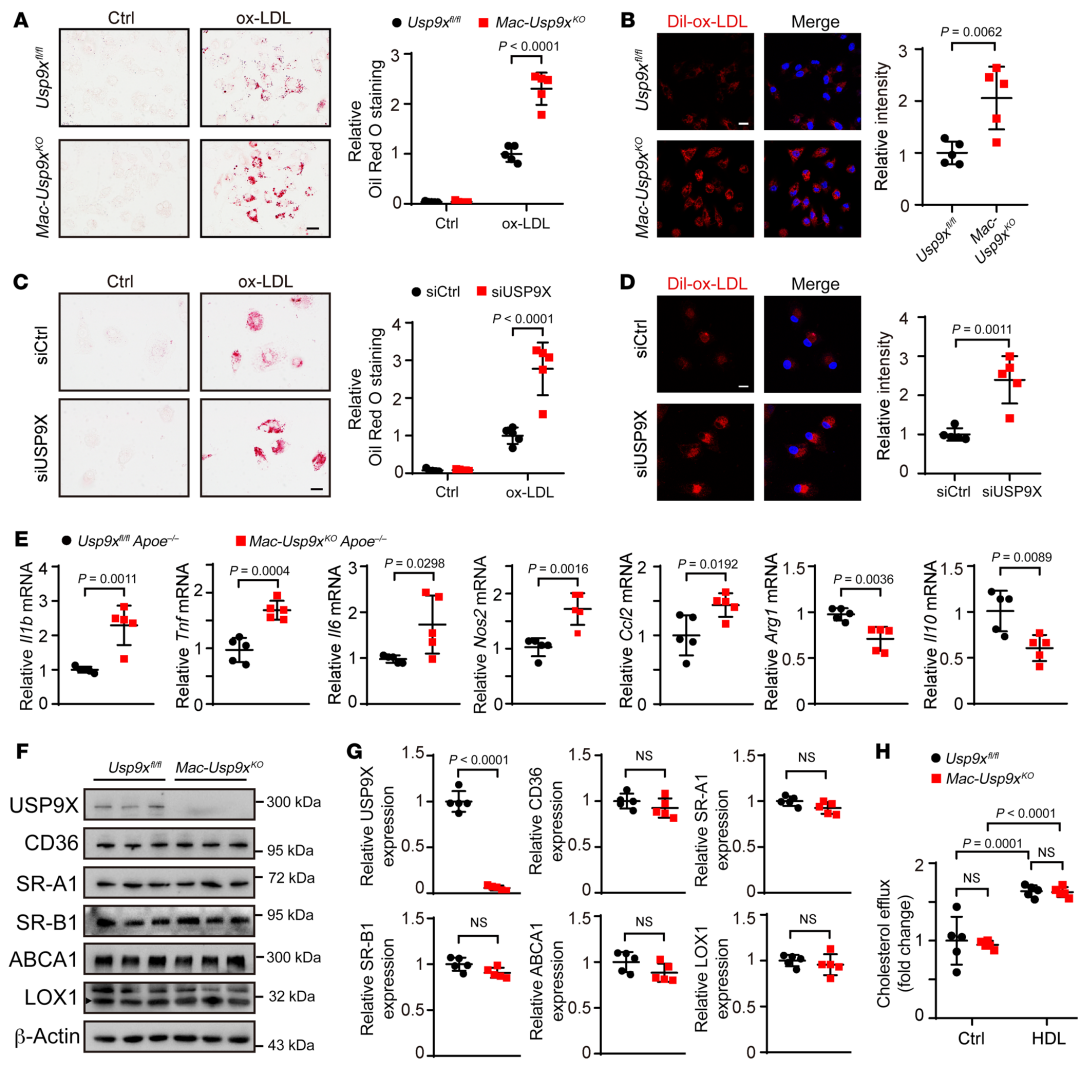
Macrophage USP9X deficiency promotes foam cell formation. (**A**) Oil Red O staining of bone marrow–derived macrophages (BMDMs) isolated from *Usp9x^fl/fl^* and *Mac Usp9x^KO^* mice and incubated with or without ox-LDL (50 μg/mL) for 24 hours. Scale bar: 20 μm. Two-way ANOVA with Bonferroni’s post hoc test (*n =* 5). (**B**) BMDMs were elicited from *Usp9x^fl/fl^* and *Mac Usp9x^KO^* mice and treated with Dil-ox-LDL (30 μg/mL) for 4 hours. Scale bar: 10 μm. Unpaired 2-tailed Student’s *t* test (*n =* 5). (**C**) Oil Red O staining of HMDMs transfected with *siCtrl* or *siUSP9X* for 48 hours and incubated with ox-LDL for a further 24 hours. Scale bar: 20 μm. Unpaired 2-tailed Student’s *t* test (*n =* 5). (**D**) Representative images of Dil-ox-LDL uptake in HMDMs transfected with *siCtrl* or *siUSP9X* for 48 hours. Scale bar: 10 μm. Unpaired 2-tailed Student’s *t* test (*n =* 5). (**E**) Plaque macrophages were isolated from aorta of *Usp9x^fl/fl^ Apoe^–/–^* or *Mac Usp9x^KO^ Apoe^–/–^* mice fed a Western diet (WD) for 12 weeks. Quantitative PCR was performed to detect the mRNA levels of the indicated genes. Target gene expression was normalized to the level of *Actb* mRNA. Unpaired 2-tailed Student’s *t* test (*n =* 5). (**F**) Western blot analysis of indicated proteins in BMDMs from *Usp9x^fl/fl^* and *Mac Usp9x^KO^* mice. (**G**) Quantification of **F**. Unpaired 2-tailed Student’s *t* test (*n =* 5). (**H**) Cholesterol efflux assay of BMDMs isolated from *Usp9x^fl/fl^* and *Mac Usp9x^KO^* mice. Two-way ANOVA with Bonferroni’s post hoc test (*n =* 5).

To further evaluate the effect of USP9X on macrophage function, we isolated BMDMs from *Mac Usp9x^KO^* and *Usp9x^fl/fl^* mice (Supplemental Figure 4A). *Usp9x*-deficient macrophages showed more esterified cholesterol accumulation and Dil-ox-LDL uptake compared with that in the control BMDMs (Figure 3, A and B). A similar trend was observed in HMDMs with siRNA-mediated silencing of *USP9X* compared with the control HMDMs (Figure 3, C and D). Furthermore, *USP9X*-deficient HMDMs showed more free cholesterol accumulation compared with that in the control macrophages (Supplemental Figure 4B). Using confocal microscopy, we observed an increase in Dil-labeled ox-LDL uptake in HMDMs and BMDMs with pharmacological inhibition of USP9X by WP1130 (Supplemental Figure 4, C and D). These findings suggest that USP9X inhibition regulates macrophage foam cell formation by increasing lipid uptake and accumulation.

Foam cell formation by infiltrating macrophages increases the production of inflammatory factors, causing instability of atherosclerotic plaques (2, 26, 27). To investigate the role of USP9X in the production of inflammatory factors by infiltrating macrophages, we isolated macrophages from the aorta of *Usp9x^fl/fl^ Apoe^–/–^* or *Mac Usp9x^KO^ Apoe^–/–^* mice fed a WD for 12 weeks. Macrophages in lesions from *Mac Usp9x^KO^ Apoe^–/–^* mice had increased expression of proinflammatory genes, such as *Tnf*, *Il6*, *Ccl2*, and *Nos2*, compared with those from control mice, while the expression of the antiinflammatory markers *arginase 1* (*Arg1*) and *Il10* was decreased (Figure 3E). At the same time, *USP9X*-deficient HMDMs had increased expression of the proinflammatory genes compared with control macrophages in response to TNF-α, while the expression of the antiinflammatory markers was decreased (Supplemental Figure 4E).

An imbalance between lipid uptake and cholesterol efflux leads to lipid accumulation in macrophages and foam cell formation. Modified lipoproteins are mainly recognized and taken up by macrophage scavenger receptors, such as CD36, SR-A1, and LOX-1. Cholesterol efflux is regulated by ATP-binding cassette transporter A1 (ABCA1), ATP-binding cassette transporter G1 (ABCG1), and other mechanisms, including SR-B1 and aqueous diffusion (28). To further explore the mechanism by which foam cell formation is increased in macrophages lacking USP9X, we analyzed the expression of scavenger receptors, ABCA1, and ABCG1 in USP9X-deficient and control macrophages. USP9X deficiency did not affect the protein or mRNA levels of the 4 scavenger receptors, ABCA1, and ABCG1 in BMDMs (Figure 3, F and G, and Supplemental Figure 4, F and G). In addition, the cholesterol efflux was comparable between the two groups (Figure 3H). These findings suggest that macrophage USP9X might modulate the function of lipid uptake–associated proteins.

### USP9X deubiquitinates the K63-linked polyubiquitin chains from SR-A1 at lysine 27.

**Figure 4:**
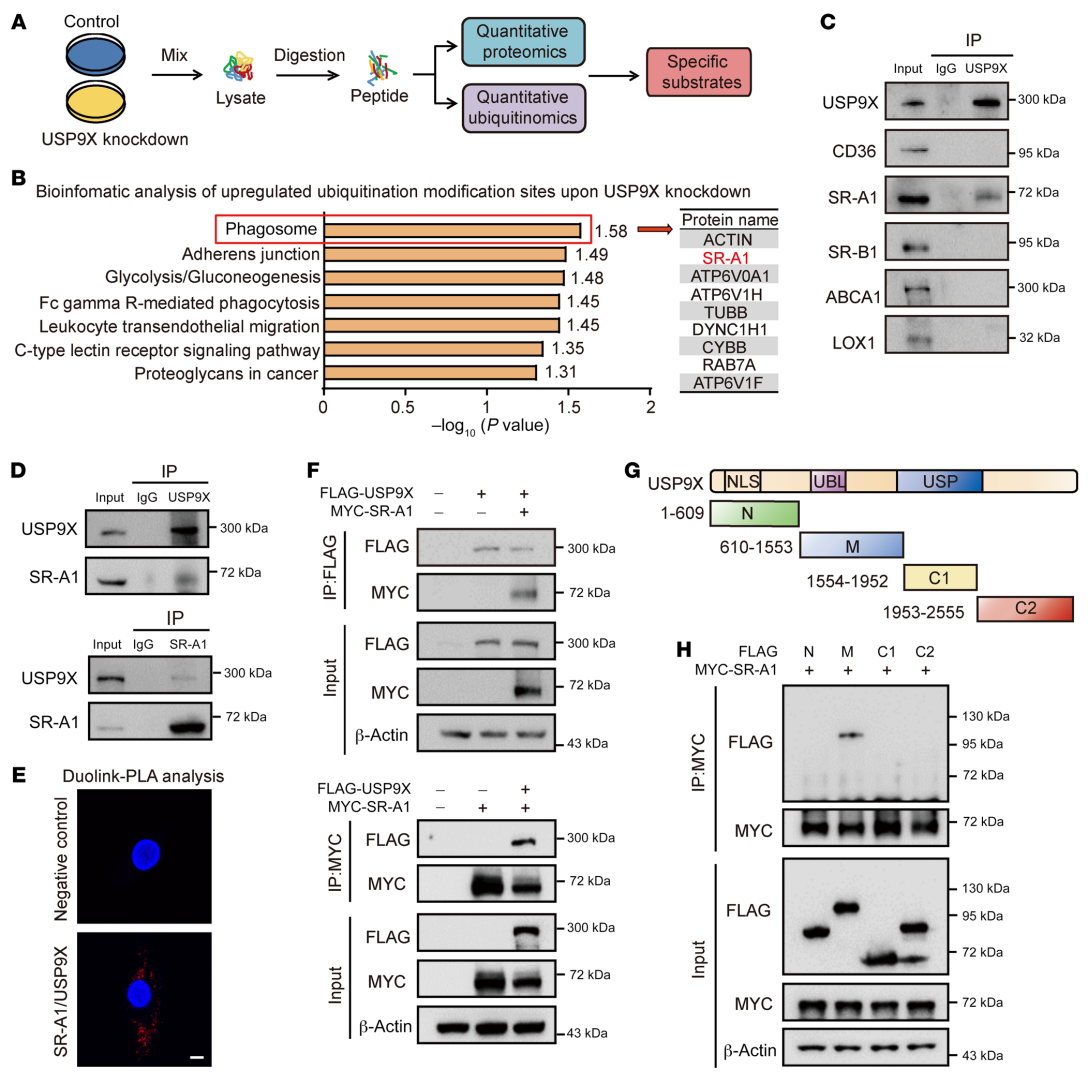
USP9X interacts directly with SR-A1. (**A**) Label-free–based quantitative proteomics and ubiquitination-modified proteomics of peritoneal macrophages transfected with *siCtrl* or *siUsp9x* for 72 hours. (**B**) KEGG pathway analysis of the proteins with significant upregulation of ubiquitinated sites. (**C**) Western blot analysis of the indicated proteins in whole cell lysates of BMDMs immunoprecipitated with anti-USP9X or rabbit IgG antibody (*n =* 5). (**D**) Western blot analysis of indicated proteins in whole cell lysates of RAW264.7 cells immunoprecipitated with anti-USP9X/SR-A1 or rabbit/mouse IgG antibody and then immunoblotted with anti–SR-A1 or USP9X antibody. (**E**) In situ proximity ligation assay (PLA) performed with proximity probes against USP9X and SR-A1 in HMDMs. Negative technical control omitting one of the primary antibodies. Nuclei were stained with DAPI (blue), and in situ PLA signals (red) indicated USP9X–SR-A1 interactions. Scale bar: 5 μm (*n =* 5). (**F**) Co-IP analysis of association of USP9X with SR-A1 in HeLa cells stably expressing doxycycline-induced FLAG-USP9X and cotransfected with MYC-SR-A1. (**G**) Schematic diagram of 4 truncation mutants of USP9X. (**H**) Western blot analysis of the indicated proteins in HEK293 cells cotransfected with constructs expressing the N/M/C1/C2 motif of USP9X and MYC-SR-A1 before IP of whole cell lysates with MYC magnetic beads (*n =* 5).

Quantitative ubiquitination site profiling using label-free quantitative proteomics was performed to identify the substrate of USP9X involved in foam cell formation in macrophages transfected with *Usp9x* or control siRNA (Figure 4A). A total of 4864 ubiquitination modification sites were identified, with quantitative information obtained for 4047 of these sites. Based on a fold-change of 2 or more in the relative amounts of differentially modified peptides between the two groups, we identified 132 significantly upregulated ubiquitination modification sites in the *Usp9x* knockdown macrophages compared with the control group. To gain insights into their biological functions, we performed a bioinformatics enrichment analysis of these proteins with upregulated ubiquitination modification sites using the Kyoto Encyclopedia of Genes and Genomes (KEGG) database. This analysis showed that these potential substrates contribute to various signaling pathways associated with processes, such as phagocytosis, adherent junction, and glycolysis. Notably, in the cluster labeled as phagosome, the ubiquitination site of the scavenger receptor SR-A1, which mediates the internalization of ox-LDL in macrophages, was more highly upregulated (Figure 4B). Therefore, we speculated that SR-A1 acts as a substrate of USP9X to mediate lipid uptake in macrophages.

Total proteins were extracted from BMDMs and immunoprecipitated with USP9X antibody that detects the endogenous proteins to confirm the interaction between USP9X and SR-A1 (Figure 4C). In contrast, other proteins mediating lipid uptake and cholesterol efflux, such as CD36, LOX-1, SR-B1 and ABCA1, were not found to bind with USP9X in BMDMs (Figure 4C). Co-IP with antibodies against USP9X or SR-A1 followed by immunoblotting with antibodies again demonstrated that these two proteins were efficiently coimmunoprecipitated (Figure 4D). In addition, immunofluorescence staining and proximity ligation assays confirmed the interaction of USP9X and SR-A1 in HMDMs (Figure 4E and Supplemental Figure 5, A and B). Moreover, we generated a HeLa cell line with doxycycline-inducible expression of stably integrated FLAG-USP9X and overexpressed MYC-tagged SR-A1. The association between FLAG-USP9X and MYC-SR-A1 was also detected in these cells (Figure 4F). To map their interaction domains, we truncated USP9X into 4 fragments: the N-terminus containing the α-α supercoil structure (N), the middle region of USP9X (M), C1 containing the ubiquitin-specific protease domain, and C2 (Figure 4G). Each of the 4 FLAG-tagged fragments were transfected into HEK293 cells along with MYC-tagged SR-A1. IP analysis revealed that the M domain of USP9X interacted with SR-A1, whereas the other 3 fragments did not (Figure 4H).

**Figure 5:**
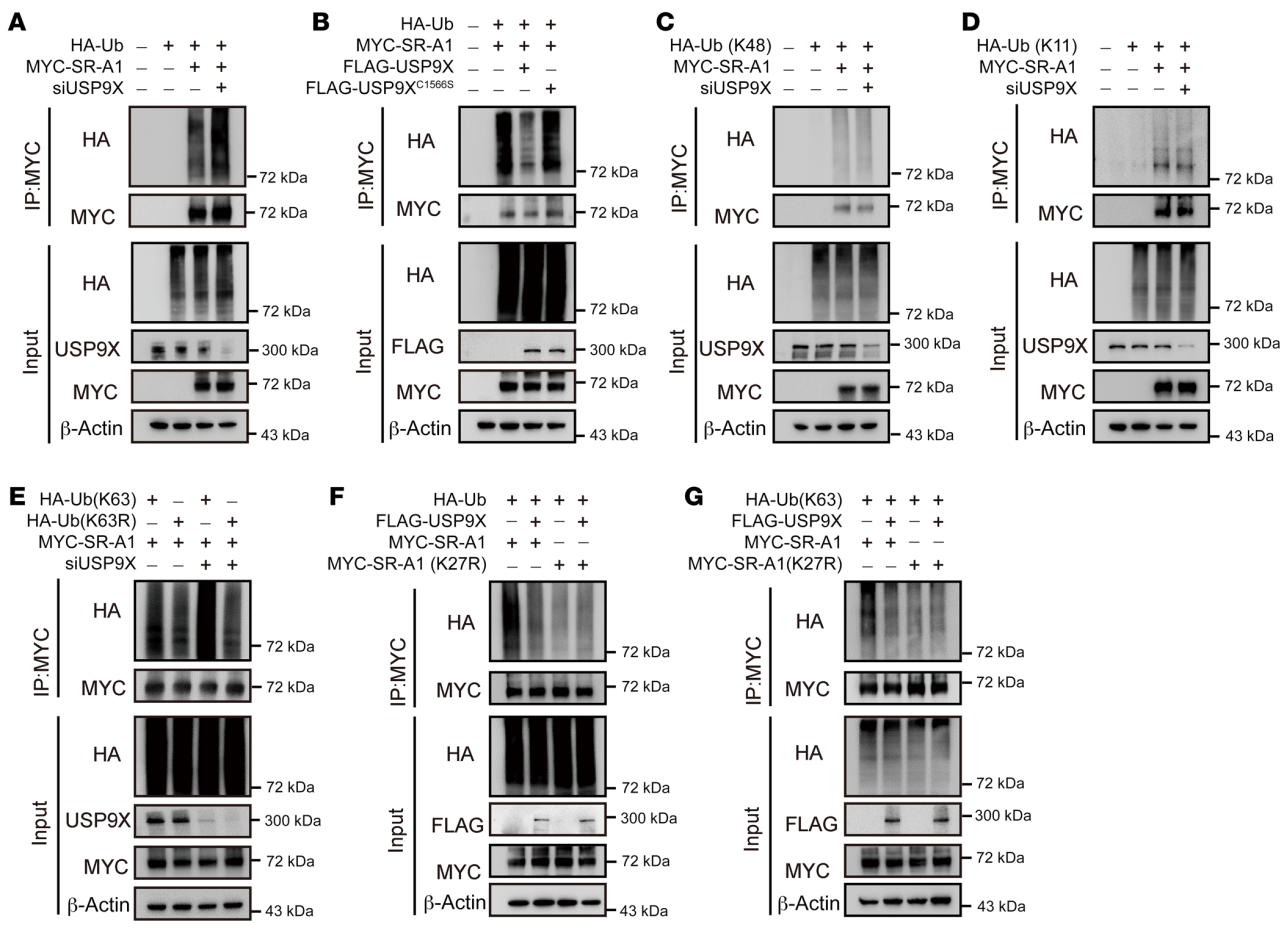
USP9X regulates K63 ubiquitination of SR-A1 at residue Lys27. (**A**) Western blot analysis of indicated proteins in HEK293 cells cotransfected with MYC-labeled SR-A1 and HA-Ub in the presence of si*Ctrl* or si*USP9X* plus the proteasome inhibitor MG132 (10 μM) for 2 hours before IP of whole cell lysates with MYC magnetic beads (*n =* 5). (**B**) Western blot analysis of indicated proteins in HeLa cells stably expressing doxycycline-induced USP9X-WT or USP9X-C1566S and cotransfected with HA-Ub and MYC-SR-A1 for 24 hours followed by induction with doxycycline (1 μg/mL) for another 24 hours and treatment with MG132 for 2 hours before IP of whole cell lysates with MYC magnetic beads (*n =* 5). (**C** and **D**) Western blot analysis of the indicated proteins in HEK293 cells cotransfected with MYC-labeled SR-A1 and HA- K11-Ub or HA-K48-Ub in the presence of si*Ctrl* or si*USP9X* plus MG132 for 2 hours before IP of whole cell lysates with MYC magnetic beads (*n =* 5). (**E**) Western blot analysis of the indicated proteins in HEK293 cells cotransfected with indicated plasmids and siRNAs plus MG132 for 2 hours before IP of whole cell lysates with MYC magnetic beads (*n =* 5). (**F** and **G**) Western blot analysis of the indicated proteins in HeLa cells stably expressing doxycycline-induced USP9X cotransfected with HA-Ub or HA-K63-Ub in the presence of MYC-SR-A1 or MYC-SR-A1-K27R for 24 hours followed by induction with doxycycline (1 μg/mL) for another 24 hours and treatment with MG132 for 2 hours before IP of whole cell lysates with MYC magnetic beads (*n =* 5).

To test whether USP9X modulates SR-A1 ubiquitination, we first overexpressed MYC-tagged SR-A1 and HA-tagged ubiquitin protein in HEK293 cells transfected with control or *USP9X* siRNA. Western blot analysis showed higher levels of total SR-A1 ubiquitination in *USP9X*-deficient cells compared with that in control cells (Figure 5A). In accordance with the effect of USP9X deficiency, pharmacological inhibition of USP9X by WP1130 increased the polyubiquitination of SR-A1 (Supplemental Figure 5C). In contrast, disruption of USP9X had no influence in the ubiquitination of CD36, another important scavenger receptor for ox-LDL (Supplemental Figure 5D). To determine whether the DUB activity of USP9X affects its regulation of the level of SR-A1 ubiquitination, we generated HeLa cell lines stably expressing doxycycline-inducible WT USP9X (USP9X-WT) or a catalytically inactive mutant of USP9X (USP9X-C1566S). The total level of SR-A1 ubiquitination was lower in cells expressing USP9X-WT than in cells expressing USP9X-C1566S induced by doxycycline (Figure 5B).

The catalytic domain of USP9X is preferentially active against K11-linked followed by K63- and K48-linked polyubiquitin chains (29). We used ubiquitin protein mutants with all lysine residues except K48, K11, or K63 replaced by arginine to identify the ubiquitin chain type of SR-A1 removed by USP9X. K63-linked polyubiquitin chains regulate protein activity, localization, and interaction with other proteins, while other polyubiquitin linkages, particularly K11- and K48-linked ubiquitin chains, target proteins for proteasomal degradation (30). In accordance with the level of intact SR-A1 protein, Western blot analysis showed that the K63-linked but not K11/K48-linked ubiquitin chain was the major form removed by USP9X (Figure 5, C and D, and Supplemental Figure 5E). In contrast, cells expressing K63R ubiquitin mutants did not respond to USP9X knockdown (Figure 5E). Together, these observations indicate that USP9X specifically removes K63-linked polyubiquitination of SR-A1.

The quantitative ubiquitination-modified proteomics analysis showed ubiquitination of 3 lysine residues K3, K27, and K42 of SR-A1 under basal conditions, and only the evolutionarily conserved K27 site ubiquitination modification was increased by siRNA-mediated inhibition of USP9X (Supplemental Figure 5F). We constructed a plasmid for SR-A1-K27R, a mutant of SR-A1 with lysine residues replaced by arginine, to block its ubiquitination. The total and K63-linked polyubiquitination of SR-A1-K27R were significantly lower than WT SR-A1 and were not further reduced by USP9X overexpression in HeLa cell lines stably expressing doxycycline-induced USP9X (Figure 5, F and G). Therefore, these findings indicate that USP9X targeted K63 polyubiquitination of SR-A1 at the K27 site.

### Mutation of the ubiquitylation site K27 of SR-A1 reduces foam cell formation and proinflammatory cytokine production.

**Figure 6:**
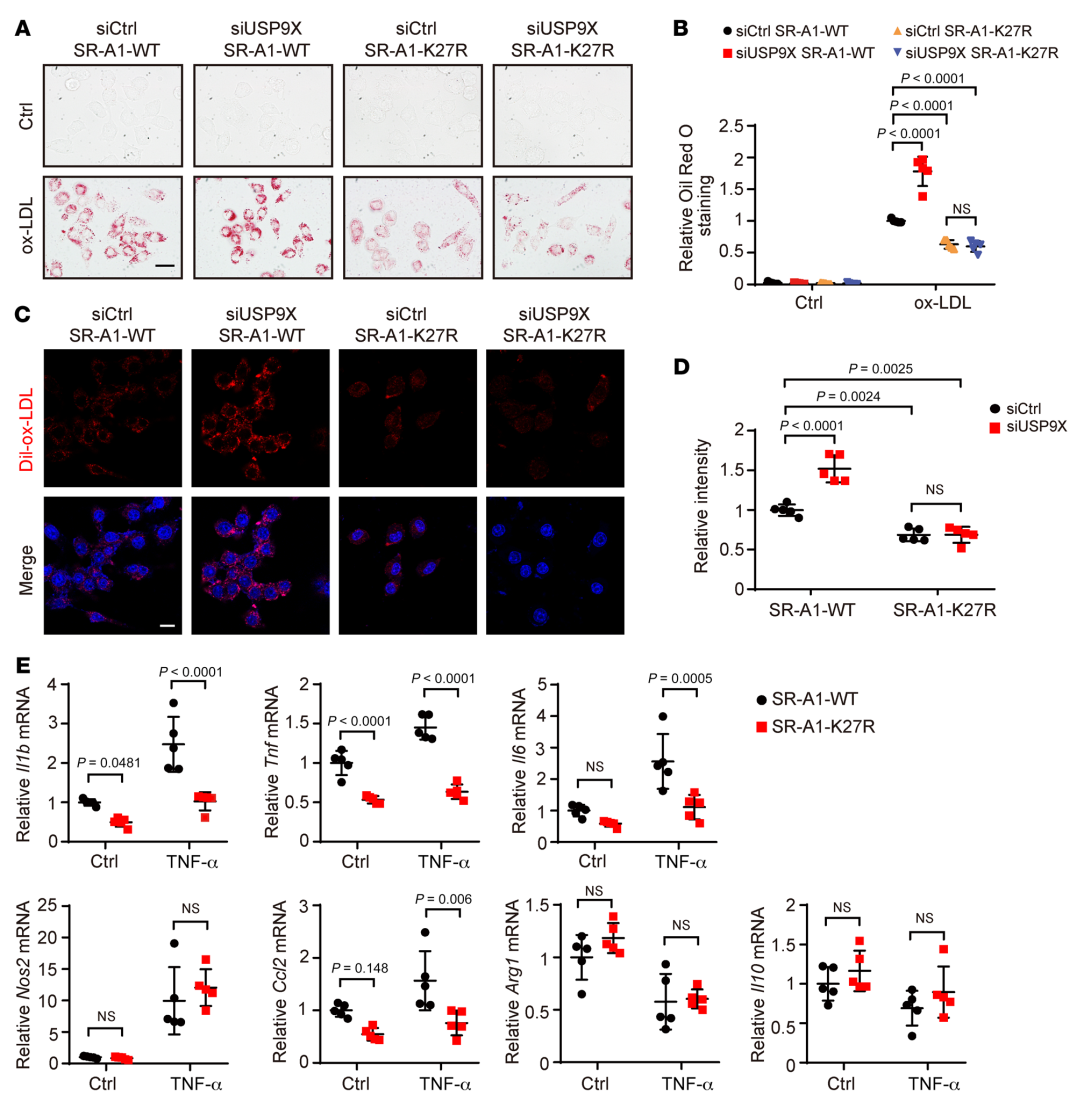
Mutation of the ubiquitylation site K27 of SR-A1 reduces foam cell formation and pro-inflammatory gene expression. (**A**) Oil Red O staining of RAW264.7 cells stably overexpressing SR-A1-WT-EGFP or SR-A1-K27R-EGFP and transfected with siCtrl or si*Usp9x* for 48 hours followed by treatment with ox-LDL for 24 hours. Scale bar: 20 μm. (**B**) Quantification of intensity of staining in **A**. Two-way ANOVA with Bonferroni’s post hoc test (*n =* 5). (**C**) Representative images of Dil-ox-LDL uptake in RAW264.7 cells stably overexpressing SR-A1-WT or SR-A1-K27R and transfected with siCtrl or si*Usp9x* for 48 hours followed by treatment with Dil-ox-LDL (30 μg/mL) for 4 hours. Scale bar: 10 μm. (**D**) Quantification of Dil-ox-LDL fluorescence intensity. Two-way ANOVA with Bonferroni’s post hoc test (*n =* 5). (**E**) Quantitative PCR analysis of indicated gene expression in RAW264.7 cells stably overexpressing SR-A1-WT or SR-A1-K27R and treated with PBS or TNF-α (30 ng/mL) for 24 hours. Target gene expression was normalized to *Actb* mRNA levels. Two-way ANOVA with Bonferroni’s post hoc test (*n =* 5).

K27 is located in the intracellular domain of SR-A1, which is essential for internalization of modified lipoproteins. Truncation of the first 27 amino acids in the cytoplasmic domain of SR-A1 reduces the uptake of Dil-Ac-LDL compared with WT SR-A1 (12). Therefore, we hypothesized that SR-A1 ubiquitination at K27 affects the uptake of modified lipoproteins. To test this hypothesis, we generated RAW264.7 cells stably expressing the SR-A1-WT/K27R-EGFP fusion protein by recombinant lentivirus infection. Oil Red O staining indicated that stable expression of SR-A1-K27R in RAW264.7 macrophages effectively reduced foam cell formation, while *Usp9x* knockdown increased lipid accumulation in macrophages with SR-A1-WT rather than SR-A1-K27R (Figure 6, A and B). Similarly, compared with SR-A1-WT macrophages, those with a mutation at K27 of SR-A1 (SR-A1-K27R) reduced the uptake of Dil-labeled ox-LDL under basal conditions and eliminated *Usp9x* deficiency–induced ox-LDL uptake (Figure 6, C and D). Furthermore, compared with SR-A1-WT macrophages, SR-A1-K27R macrophages had lower expression of proinflammatory genes, such as *Il1b*, *Tnf*, *Il6*, *Nos2*, and *Ccl2*, whereas there was no apparent difference in the expression of the antiinflammatory genes *Il10* and *Arg1* in response to TNF-α (Figure 6E). These findings indicate that the ubiquitination of SR-A1 at K27 enhanced the uptake of modified lipoproteins, foam cell formation, and inflammatory responses.

### K63-linked ubiquitination of SR-A1 mediates its internalization by macrophages.

**Figure 7:**
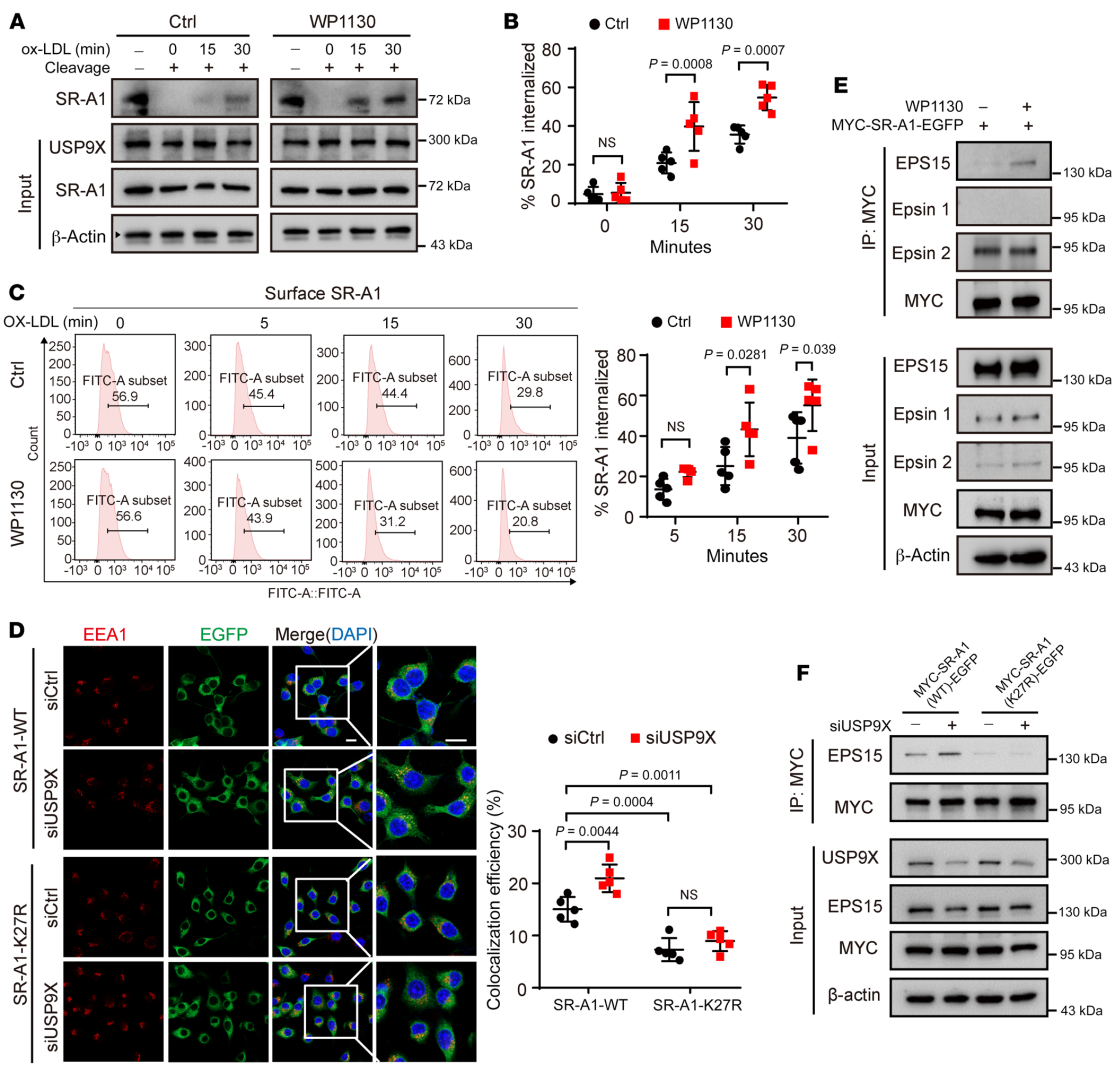
K63-linked ubiquitination of SR-A1 promotes its internalization. (**A**) Western blot analysis of internalized biotinylated proteins in BMDMs from WT mice treated with or without WP1130 for 24 hours before surface receptor biotinylation and internalization (see Supplemental Methods). (**B**) Quantification of SR-A1 internalization in **A**. Two-way ANOVA with Bonferroni’s post hoc test (*n =* 5). (**C**) Flow cytometric analysis of surface expression of SR-A1 protein by cells after ox-LDL stimulation for the indicated times. Two-way ANOVA with Bonferroni’s post hoc test (*n =* 5). (**D**) Immunofluorescence analysis of RAW264.7 cells stably overexpressing SR-A1-WT-EGFP or SR-A1-27R-EGFP and transfected with control or *Usp9x* siRNA for 48 hours before incubation with ox-LDL for 30 minutes to induce internalization. Colocalization of SR-A1 (EGFP, green) and EEA1 (red) is shown (left) and quantified (right). Two-way ANOVA with Bonferroni’s post hoc test (*n =* 5). Scale bar: 10 μm. (**E**) Western blot analysis of the indicated proteins in RAW264.7 cells stably overexpressing SR-A1-WT-EGFP and treated with or without WP1130 for 24 hours before incubation with ox-LDL for 30 minutes to induce internalization and IP of whole cell lysates with MYC magnetic beads (*n =* 5). (**F**) Western blot analysis of the indicated proteins in RAW264.7 cells stably overexpressing SR-A1-WT-EGFP or SR-A1-K27R-EGFP and transfected with siCtrl or si*Usp9x* for 48 hours before incubation with ox-LDL for 30 minutes to induce internalization and IP of whole cell lysates with MYC magnetic beads (*n =* 5).

To determine whether USP9X affects lipid uptake by regulating SR-A1 internalization, we analyzed the levels of internalized SR-A1 protein in macrophages with genetic or pharmacological inhibition of USP9X. In an internalization assay, ox-LDL–induced SR-A1 internalization was first detected in the first 15 minutes after stimulation. From 15 to 30 minutes, faster kinetics of ox-LDL–induced SR-A1 internalization was observed in WP1130 or *Usp9x*-deficient macrophages compared with each control group, although the total cellular content of the receptor remained unchanged (Figure 7, A and B, and Supplemental Figure 6, A and B). The internalization of SR-A1 stimulated by ox-LDL was also evaluated by flow cytometry. SR-A1 was internalized more rapidly in WP1130-treated macrophages after ox-LDL stimulation than in the control group (Figure 7C). To determine whether *Usp9x* deficiency–induced SR-A1 ubiquitination at K27 contributes to SR-A1 internalization in endosomes, we transfected *Usp9x* or control siRNA in macrophages expressing SR-A1-WT-EGFP or SR-A1-K27R-EGFP. The localization of SR-A1 was detected by the intrinsic fluorescence of EGFP, and the early endosome marker early endosome antigen 1 (EEA1) or the late endosome/lysosome marker (LAMP1) was localized by indirect immunofluorescence with anti-EEA1 or anti-LAMP1 antibodies. After 30 minutes of continuous phagocytosis of lipoproteins, confocal fluorescence microscopy showed that a portion of SR-A1-WT colocalized with EEA1. Compared with the control, *Usp9x* knockdown increased the colocalization of SR-A1 and EEA1. In contrast, the colocalization of SR-A1-27R and EEA1 was much less marked than that of SR-A1-WT and was not enhanced by *Usp9x* knockdown (Figure 7D). After 1 hour, only a very small amount of SR-A1 colocalized with LAMP1, and this was not affected by *Usp9x* deficiency or mutation of SR-A1 at K27 (Supplemental Figure 6, C and D).

Clathrin-dependent endocytosis requires endocytic adaptors, such as Eps15 and Epsin1/2 (31), which usually contain a ubiquitin-interacting motif (UIM) or ubiquitin-related domain (UBA). These adaptors recognize and bind ubiquitin to mediate internalization of the ubiquitinated receptor (32). Our results showed that after inhibiting USP9X, the combination of SR-A1 with Eps15, but not Epsin1 or Epsin2, was increased during ox-LDL–mediated internalization (Figure 7E). In addition, SR-A1-27R bound to Esp15 to a much lesser extent than SR-A1-WT in response to ox-LDL stimulation, which was regulated by USP9X inhibition (Figure 7F). Although Eps15 was identified as a USP9X substrate in tumors (18), we did not observe the change of level of EGFR pathway substrate 15 (EPS15) ubiquitination in macrophages with siRNA-mediated *Usp9x* knockdown (Supplemental Figure 6E). These findings suggest that *Usp9x* deficiency–induced SR-A1 ubiquitination at the K27 site facilitated the internalization of SR-A1 and ox-LDL uptake by enhancing the recognition and binding of the cytoplasmic domain of SR-A1 by Esp15.

### Blockade of the USP9X–SR-A1 interaction promotes foam cell formation and atherosclerosis.

**Figure 8:**
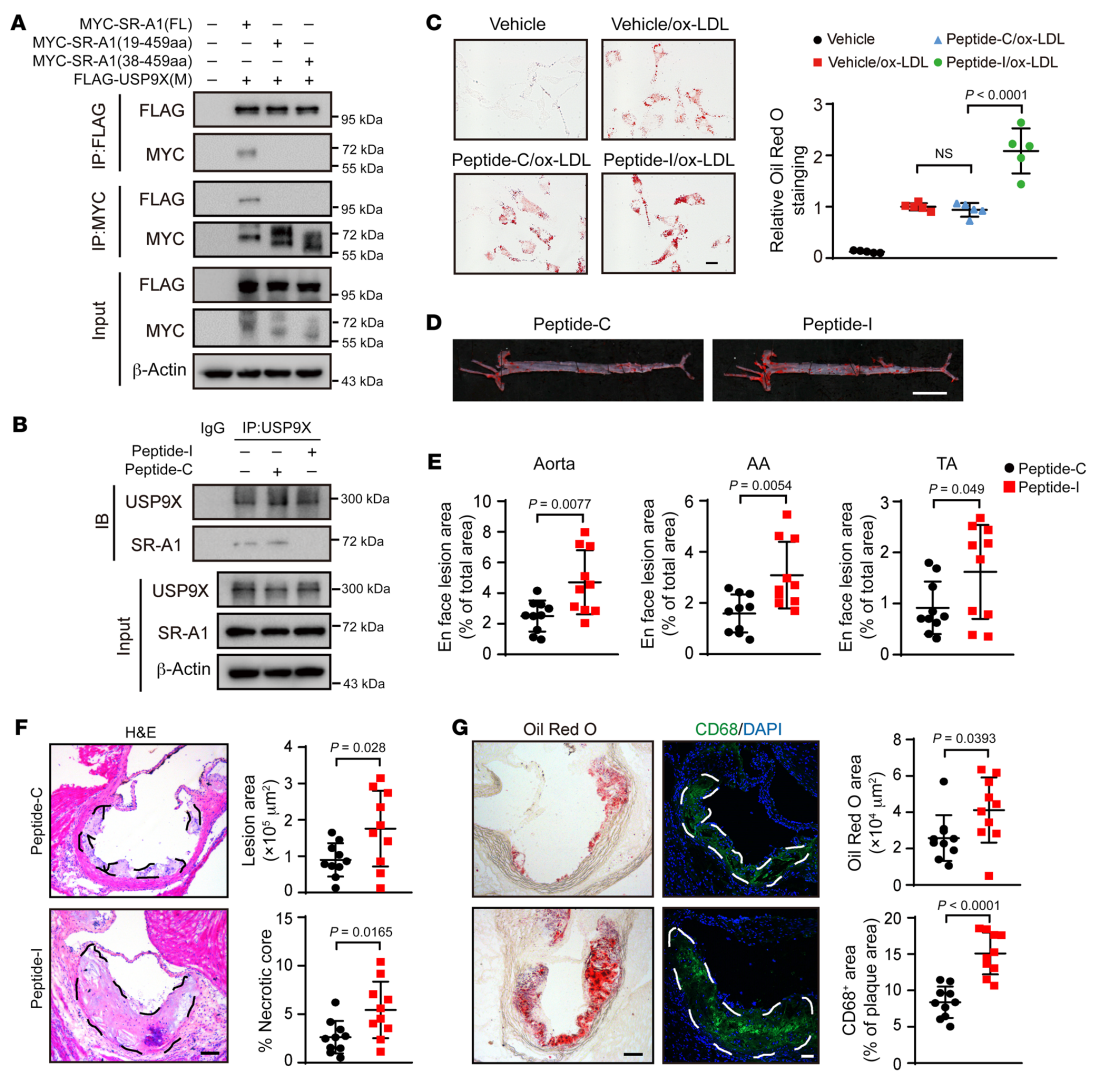
An inhibitory peptide blocks the SR-A1–USP9X interaction and promotes foam cell formation and atherosclerosis. (**A**) HEK293 cells were cotransfected with constructs expressing the middle region of USP9X (FLAG-USP9X-M) and MYC tagged different truncations of the intracellular segment of SR-A1 before IP of whole cell lysates with FLAG magnetic beads (*n =* 5). (**B**) BMDMs were treated with different peptides (20 μM) for 24 hours (*n =* 5). The cells were subjected to IP with antibody against USP9X or IgG to quantify the interaction of USP9X and SR-A1. (**C**) Oil Red O staining analysis was performed. (**D**) Oil Red O staining of aortas from control or inhibitory peptides administered to *Apoe^–/–^* mice fed WD for 10 weeks (*n =* 10). Scale bar: 5 mm. (**E**) Quantification of plaque area/total vessel area. AA, aortic arch; TA, thoracic aorta. Unpaired 2-tailed Student’s *t* test (*n =* 10). (**F**) Representative images of H&E staining of aortic root sections (left) and quantification of lesion and necrotic core areas (right). Black dashed lines demarcate atherosclerotic plaques. Scale bar: 100 μm. Unpaired 2-tailed Student’s *t* test (*n =* 10). (**G**) Oil Red O (left) staining of aortic root sections. Scale bar: 100 μm. Macrophages identified by anti-CD68 antibody staining (right). The white dashed line indicates plaques. Scale bar: 50 μm. Unpaired 2-tailed Student’s *t* test (*n =* 10).

To further investigate the function of the interaction of USP9X with SR-A1, we used truncated forms of the intracellular segment of SR-A1 and revealed that SR-A1 aa 1–18 was essential for its binding to USP9X (Figure 8A). Based on this result, we synthesized the corresponding cell-penetrating inhibitory peptide (peptide-I) and studied the effects of blocking the combination of SR-A1 and USP9X on foam cell formation and atherosclerosis in vitro and in vivo. We found that peptide-I, but not the control peptide (peptide-C), abolished the interaction between USP9X and SR-A1 in macrophages (Figure 8B). In addition, macrophages exposed to peptide-I exhibited more ox-LDL–induced lipid droplet formation and uptake of Dil-labeled ox-LDL compared with those treated with peptide-C (Figure 8C and Supplemental Figure 7A). We treated WD-fed *Apoe^–/–^* mice with the peptides at 20 mg/mL for 10 weeks and investigated the impact on atherosclerosis (Supplemental Figure 7B). Similar to mice with macrophage *Usp9x* deficiency, administration of peptide-I dramatically increased the atherosclerotic lesion areas in the whole aorta and aortic roots compared with the effects of peptide-C (Figure 8, D–F). Furthermore, exposure to peptide-I led to 57% and 80% increases in Oil Red O– and CD68-positive areas, respectively, compared with mice treated with peptide-C (Figure 8G). The body weight and plasma lipid levels were comparable between the two groups (Supplemental Figure 7C). Thus, these results suggested that peptide-I increased macrophage foam cell formation and atherosclerosis progression by disrupting the interaction between USP9X and SR-A1, which mimicked the proatherosclerotic effect of USP9X deficiency.

## Discussion

In this study, we used in vitro screening of macrophages to search for DUBs that regulate foam cell formation. USP9X-deficient macrophages were found to enhance lipid uptake and lipid accumulation in response to ox-LDL. Compared with mild atherosclerotic plaque, USP9X protein levels were downregulated in severe plaques. In mouse models, macrophage-specific *Usp9x* deficiency increased lipid deposition, lesional macrophage content, and necrotic cores. Lesional macrophages from *Mac Usp9x^KO^ Apoe^–/–^* mice had higher proinflammatory gene expression compared with control mice. In terms of the mechanism, we discovered that the antiatherosclerotic effect of USP9X was attributable to the regulation of SR-A1 internalization, thereby reducing foam cell formation. Therefore, our research defined the functional role of macrophage USP9X in atherosclerosis and highlights potentially new strategies for treating this disease.

In this study, we showed that the protein level of macrophage USP9X was gradually reduced with the continuation of WD feeding time. However, the mRNA levels of macrophage *Usp9x* were comparable. Several factors might contribute to this phenomenon. Noncoding RNAs have been shown to regulate gene expression through translational repression. For instance, miR-206 and miR-29 control myogenic differentiation through translational repression of HDAC4 (33). In addition, miR455-3p binds to the 3′-UTR of HSF1 mRNA and can repress its translation (34). Long non-coding RNAs, including LincRNA-p21 and lncRNA GAS5, have also been reported to pair with target RNAs to inhibit translational efficiency (35, 36). N6-methyladenosine, the most abundant internal modification in mammalian mRNA, adds another layer to the dynamic control of the translational machinery and protein production. These mechanisms may underlie the regulation of USP9X expression and protein production in macrophages isolated from WD-fed mice, and further studies are warranted to explore this issue.

Excessive uptake of intracellular lipids is one of the main reasons for foam cell formation (8), which is a hallmark of atherosclerosis progression. Modified lipoproteins are recognized by transmembrane scavenger receptors expressed by macrophages, which are subsequently ubiquitinated and mediate internalization of the cargo protein. This process requires the ubiquitin-binding adaptor protein, which binds ubiquitin via a UIM or UBA domain. Several ubiquitin-binding adaptor proteins, such as epsin 1, epsin 2, and EPS15, have been identified, all of which belong to the clathrin-related sorting protein (CLASP) family (37–39). USP9X has been implicated in regulating endocytosis. USP9X has been reported to simultaneously regulate endocytosis of the EGFR by deubiquitinating its endocytic adaptor Eps15 and ubiquitin ligase Itch in tumor cell lines (17, 18, 40). In contrast, USP9X also binds directly to receptors such as ErbB2/HER2, thereby affecting the ubiquitination status of the receptors and regulating their intracellular localization and transport (41). This is consistent with our finding that USP9X interacts physically with and deubiquitinates SR-A1, thereby reducing internalization of the ox-LDL-SR-A1 complex and subsequent foam cell formation. We showed that the K63 ubiquitination of SR-A1 promotes its internalization by recruiting Eps15. Interestingly, the ubiquitination of ESP15 in macrophages was intact under conditions of USP9X depletion, suggesting that USP9X targets substrates specifically in distinct cell types. However, because of the involvement of other ubiquitin-binding adaptors in endocytosis (42), we cannot exclude the effect of other adaptors on its internalization following recognition of ubiquitinated SR-A1.

As one of the main scavenger receptors, SR-A1 is responsible for the uptake of modified lipoproteins by macrophages and mediates the formation of foam cells (43, 44). SR-A1–mediated internalization of lipoproteins relies on clathrin-dependent endocytosis, a major membrane receptor internalization process. After internalization into endosomes, the ligand dissociates from the receptor and is transported from the endosome to the lysosome for metabolization, while the receptor is recycled to the cell surface (45). The cytoplasmic domain of SR-A1 plays an important role in membrane transport, recycling, and internalization (11). Mutants of membrane-proximal amino acids SR-A1^Δ1-49^ cannot internalize lipoproteins. In addition, removing the first 27 amino acid residues from the N-terminus of SR-A1 also reduces cellular uptake of lipoproteins (12, 13). In this study, we discovered that USP9X removed K63 polyubiquitination at the K27 site of SR-A1. It has been reported that receptor internalization and sorting after endocytosis are regulated by K63 polyubiquitination modification (46, 47). Indeed, we found that K63 polyubiquitination at the K27 site of SR-A1 increased its internalization with ox-LDL. Macrophage SR-A1 promotes atherosclerosis by facilitating inflammation and apoptosis (48). The K63 polyubiquitination of SR-A1 recruits the TAK1/MKK7/JNK signaling complex, and activation of the JNK signaling pathway promotes the conversion of macrophages to proinflammatory types (49). Moreover, SR-A1 activation leads to activation of the NF-κB signaling pathway and affects the conversion of foamy macrophages to a proinflammatory phenotype (50). Notably, regions with macrophages expressing high levels of proinflammatory factors, such as IL-1β, IL-6, and TNF-α, are more susceptible to rupture and become unstable plaques by promoting cell death and reducing the plaque intensity (51–54). Our observations revealed that macrophages with mutant SR-A1-K27R expressed much lower levels of proinflammatory factors than those expressing SR-A1-WT. This evidence indicates that SR-A1 ubiquitination promotes the internalization of modified lipoproteins by macrophages and leads to the generation of a proinflammatory phenotype, a transformation that synergistically accelerates plaque foam cell formation and plaque instability. Interestingly, a recent single-cell RNA sequencing study in a murine model of atherosclerosis showed that nonfoamy macrophages have proinflammatory characteristics, while foamy macrophages do not (5). Thus, the origin, drivers, and dynamics of this heterogeneity in plaque macrophages as well as the function of USP9X in them should be explored in the future.

USP9X dysfunction contributes to neurological diseases and cancers (55–57). WP1130 has been shown to increase the sensitivity of cancer cells, such as hepatocellular carcinoma cells, breast cancer cells, and pancreatic cancer cells, to chemotherapeutic agents by inhibiting USP9X activity (58–60), although no relevant clinical trials have been reported to date. These studies indicate the value of USP9X as a potential therapeutic target in cancer. However, our findings raise the possibility that USP9X inhibitors may cause side effects in the cardiovascular system by inducing atherosclerosis progression. Thus, combined treatment with a USP9X inhibitor and statins or anti-PCSK9 antibodies could be a new therapeutic strategy that prevents the cardiovascular side effects associated with administration of the USP9X inhibitor alone.

Some limitations of this study should be acknowledged. First, we used ox-LDL as an inducer of foam cells. Although there is compelling evidence that oxidation of LDL occurs in vivo, detailed characterization of naturally occurring ox-LDL is technically challenging and remains largely unclear. Second, noncoding RNAs, such as miRNAs and lncRNAs, have been identified as regulators of DUBs. Recent studies showed that miR-26b overexpression or transfection with miR-212 mimicked reduced USP9X expression, whereas circRNA hsa_circ_0008434 enhanced the expression of USP9X (61–63). Although the regulation of USP9X by noncoding RNAs in the context of atherosclerosis is still unknown, the current evidence suggests that it is worth investigating the underlying mechanism with a view to developing a therapeutic USP9X agonist for atherosclerosis.

In summary, our current study demonstrated that USP9X has a role in inhibiting the development of atherosclerosis. We have revealed a mechanism by which internalization of the scavenger receptor SR-A1 is regulated, whereby USP9X regulates K63 polyubiquitination at the K27 lysine residue of SR-A1 and reduces its internalization, leading to the inhibition of modified lipoprotein uptake and foam cell formation. These findings reveal the negative regulatory role of macrophage USP9X in the development of atherosclerosis and highlight potentially new strategies for the treatment of atherosclerosis.

## Methods

All supporting data are available within the article and Supplemental Methods. Detailed descriptions of experimental methods of the current study are provided in Supplemental Methods.

### Statistics.

For all the experiments, effect sizes were estimated based on preliminary data, and selected cohort sizes for all experiments were sufficient to give a power of 0.8 at an α of 0.05. Analyses were conducted in a blinded fashion. No outliers were excluded. The numbers of replicates are indicated in the figure legends. The data were tested for normality before parametric statistical analysis using the Shapiro-Wilk normality test (*n* < 10). For normally distributed data, comparisons between 2 groups were performed using unpaired or paired 2-tailed Student’s *t* test, and comparisons among multiple groups were performed using 1-way or 2-way ANOVA followed by Bonferroni’s post hoc test. Comparisons of nonnormally distributed data were performed using the Mann-Whitney *U* test. *P* values are shown in the figures. Fisher’s exact test was used to test the enrichment of differentially expressed proteins against all identified proteins in ubiquitylomics data. Adjusted *P* values of less than 0.05 were considered significant. Technical repeats, single patients, or mice are indicated by single symbols, if applicable. The statistical tests used for analysis of the data are stated in the figure legends for each experiment. GraphPad Prism (version 8.0) was used for statistical analysis. All data are presented as mean ± SEM. *P* < 0.05 was set as the threshold for statistical significance.

### Study approval.

All experiments using human being were reviewed and approved by the Ethics Committee of Tianjin Medical University General Hospital, and written, informed consent was obtained from all participants. All animal experimental protocols were conducted in accordance with the NIH *Guide for the Care and Use of Laboratory Animals* (National Academies Press, 2011) and approved by the Institutional Animal Care and Use Committee of Tianjin Medical University.

## Author contributions

BW, DA, and HJ designed the research; BW, XT, LY, YW, ZC, ML, and HJ performed the research; BW, LY, and XT analyzed the data; DA provided the experimental animals; DW, NW, and XD collected human samples; and BW and DA wrote the paper. All authors read and approved the manuscript.

## Supplementary Material

Supplemental data

## Version 1 04/07/2022

In-Press Preview

## Version 2 05/16/2022

Electronic publication
