# Supplementary material for: Disruption of USP9X in macrophages promotes foam cell formation and atherosclerosis
Source: J Clin Invest. 2022 May 16;132(10):e154217. doi: 10.1172/JCI154217 (PMC9106359; doi:10.1172/JCI154217)
Supplement: Supplemental data [file jci-132-154217-s254.pdf]

Supplemental Material

Supplemental materials & methods

Reagents

SiRNA for *Usp9x* (sc-100628) and *control* siRNA (sc-37007) were from Santa Cruz Biotechnology (Santa Cruz, CA, USA). SiRNAs for *STAMBPL3, PRPF8, A20, USP39, USP24, JOSD1, USP22, USP18, USP9X, OTUD4* were from General Biosystem (Anhui, China). TNF-α (10291-TA) was from R&D Systems. ox-LDL (JK-002) and Dil-ox-LDL (JK-015) were from Jingkehuaxue (Shanghai, China). Malondialdehyde was measured using the TBARS colorimetric method with an appropriate standard. Starting LDL <0.50 nmol of MDA/mg protein, ox-LDL >18.5 nmol of MDA/mg protein. Dox (D8740), LPS (L8880), IFNγ (P00106), puromycin (P8230), and G418 (IG0010) were from Beijing Solarbio Science & Technology Co (China). PMA (P1585) and 2-mercaptoethanol (M6250) were from Sigma-Aldrich (USA). WP1130 (S2243) and MG132 (S2619) were from Selleckchem (Selleck, TX, USA). Human/murine M-CSF (300-25/ 315-02) was purchased from Peprotech (Cranbury, NJ, USA). Antibodies against USP9X (55054-1-AP), CD36 (18836-1-AP), ABCG1 (13578-1-AP), USP39 (23865-1-AP), USP24 (13126-1-AP) were from Proteintech (Wuhan, China). Antibodies against USP9X (sc-36353 for IF), SR-A1 (sc-166139), β-actin (sc-8432), Epsin2 (sc-376788) and USP18 (sc-374064) were from Santa Cruz Biotechnology (Santa Cruz, CA, USA). Antibodies against CD68 (ab955), SR-B1 (ab217318), LOX1 (ab60178), ABCA1 (ab18180), Epsin1 (ab75879), Eps15 (ab174291) and FITC-SR-A1 (ab275706 for FACs) were from Abcam (Cambridge, UK). Antibodies against αSMA (A52285) and Flag (F1804) were from Sigma-Aldrich (USA); HA (3724). Antibodies against ubiquitin (43124), RAB7

(D95F2), EEA1 (C45B10), LAMP1 (D2D11), rabbit IgG (7074), mouse IgG (7076) and Myc (9B11) were from Cell Signaling Technology (Boston, MA, USA). Anti-SR-A1 (A14187 for IF/WB) was from ABclonal (Wuhan, China). Anti-OTUD4 (NBP1-77003) was from Novus biologicals (USA).

Clinical study participants

Atherosclerotic plaques were obtained within the same human carotid endarterectomies. All protocols were approved by the Tianjin Medical University General Hospital Ethics Committee (IRB2020-YX-074-01). Blood coagulation was blocked with EDTA and PBMCs were isolated using Histopaque-1077 as previously described (1). The baseline characteristics of the patients are shown in Tables S1. This study was performed in accordance with the Declaration of Helsinki and participants provided with informed consent.

Animals

Usp9x$^{flox/flox}$ mice were kindly provided by Dr. Stephen A. Wood (Griffith University, Brisbane, Australia). We generated myeloid cell-specific *Usp9x* knockout mice (*Mac-Usp9x*^{KO}) by crossing *Usp9x*$^{flox/flox}$ mice with transgenic mice expressing Cre recombinase under the control of a *LysM* promoter (Jackson Laboratories; stock #004781). *LysMCre-Usp9x*$^{flox/flox}$*-Apoe*$^{-/-}$ (*Mac-Usp9x*^{KO}*-Apoe*$^{-/-}$) mice were obtained by crossing apolipoprotein E-knockout (*Apoe*$^{-/-}$) mice (Jackson Laboratories; stock #002052) with the *Mac-Usp9x*^{KO} mice. Female or male mice (aged 8 weeks) were used in this study and fed Western diet (WD) (TD.88137, Harlan Teklad) for the indicated time-period. All animal experiments were approved by the Institutional Animal Care and Use Committee of the Tianjin Medical University. All mice were bred on a C57BL/6 background and maintained under a 12:12 h light/dark cycle (lights

on at 7:00 and off at 19:00) before and during experiments.

Cell culture and stably transfected cell line generation

Bone marrow cells were isolated as described previously (2), and incubated for 7 days with murine M-CSF (50 ng/ml) to induce bone marrow-derived macrophage (BMDM) differentiation. Peritoneal macrophages were harvested from mice 3 days after intraperitoneal injection of 3% thioglycolate as described previously (3). Human monocyte-derived macrophages were obtained by incubation of peripheral blood mononuclear cells with human M-CSF (50 ng/ml) for 7 days. HeLa, HEK293, HEK293T, RAW264.7 and THP-1 cells were obtained from the American Type Culture Collection (Manassas, VA, USA) and were cultured according to the supplier's instructions. All cell lines were authenticated by examination of morphology and growth characteristics, and were confirmed to be mycoplasma-free. SR-A1-WT/K27R-EGFP stably transfected RAW264.7 cells were generated using a two-step protocol. First, cells were infected with lentivirus carrying pCDH-EF1-MCS-T2A-Puro and encoding SR-A1-WT/K27R-EGFP. Subsequently, the infected cells were subjected to puromycin selection and protein expression in transfected cells was confirmed by Western blot analysis. HeLa cells stably expressing USP9X-WT/ USP9X-C1566S under DOX treatment were kindly provided by Dr. Lei Shi (Tianjin Medical University, Tianjin, China) as previously reported (4).

Human DUB siRNA library screening

The human ON-TARGET plus siRNA Library-Deubiquitinating Enzymes-SMART pool (G-104705, Dharmacon, USA) was used to screen for human DUBs. THP-1 cells were seeded in a 96-well plate containing the transfection reagent-siRNA complex (50 nM final

concentration). After 6 h, the cells were incubated with phorbol 12-myristate 13-acetate (PMA)-containing media to induce the differentiation of THP-1 cells. After 48 h, fresh medium containing ox-LDL was added and cells were incubated for a further 24 h prior to Oil Red O staining.

Protein extraction and label-free quantitative analysis of ubiquitylomes

The proteins were extracted from mouse peritoneal macrophages transfected with *Usp9x* or control siRNA, digested and affinity-enriched the ubiquitin-modified peptides. The enriched peptides were then subjected to HPLC-MS/MS analysis for ubiquitinome quantification. The cells were lysed to extract the proteins and the extraction were digested by trypsin. The ubiquitinated peptides were obtained through immunoprecipitation with anti-diglycine lysine antibody conjugated agarose beads (PTM-1104, PTM Biolabs, China). We used a label-free proteomic workflow to analyze the sequence of intact peptides (PTM Biolabs, China), and the corresponding MS/MS data were performed with MaxQuant search engine (v1.5.2.8). Tandem mass spectra were searched against a database concatenated with the reverse decoy database. The relative quantification of each sample was calculated according to the intensity of the peptides between different samples. Only if the fold change between two groups were more than 1.5, the change threshold was regarded as significant.

Immunofluorescence staining

An essential preliminary step was fixed cell or tissue slides with 4% paraformaldehyde for 30 min to preserve morphology while maintaining antigenicity, washed three times in PBS. After the above fixation step, the slides were permeabilized in PBS/0.05% Triton X-100 for 15 min, nonspecific binding site block can be achieved by incubation in a 3% bovine serum albumin

/PBS solution for 30 min at room temperature. Then slides were incubated in the primary antibody solution at 4°C overnight. After washing three times with PBS, the cells were incubated with Alexa Fluor 488 (A-10680)- or Alexa Fluor 594 (A-11037)-conjugated secondary antibodies (Thermo Fisher Scientific, MA, USA) for 2 h at room temperature. Finally, DAPI was used to cover slides. Fluorescent signals were detected by confocal laser scanning microscopy.

Quantification of lipid levels

Circulating blood from mice was collected into heparin-coated test tubes, and plasma was separated by centrifugation. Plasma triglycerides (TG), total cholesterol (CHO), low-density lipoprotein cholesterol (LDL-C) and high-density lipoprotein cholesterol (HDL-C) levels were measured by using kits (BioSino Bio-Technology and Science, Beijing, China).

Western blot analysis

Cells or tissues were homogenized in cold RIPA lysis buffer supplemented with complete protease inhibitor cocktail and phosSTOP phosphatase inhibitor (Roche, Switzerland). Equivalent levels of proteins were denatured, resolved by SDS-polyacrylamide gel electrophoresis (SDS-PAGE) and transferred to nitrocellulose membranes. Each membrane was incubated with 5% skimmed milk and probed with primary antibodies overnight at 4°C. Bound antibodies were detected by horseradish peroxidase–conjugated secondary antibody (1:5000) and visualized by enhanced chemiluminescence (KF8003, Affinity Biosciences).

Immunoprecipitation

Immunoprecipitation assay was performed as described (48). Cell lysates were prepared by incubating the cells in lysis buffer supplemented with protease inhibitor Cocktails for 30 min

at 4°C, which was followed by centrifugation at 12,000 rpm for 15 min at 4°C. Control or specific antibodies were added into cell lysates for 12 h at 4°C with constant rotation; 30 μl of pre-washed protein A/G agarose beads were then added and the incubation was continued for an additional 2 h. For magnetic beads (Bimake, China), washed beads were directly added to cell lysates, and gently rotated over night at 4°C. The precipitated proteins were eluted from the beads by re-suspending the beads in 2× SDS-PAGE loading buffer and boiling for 5 min. The boiled immune complexes were subjected to SDS-PAGE, followed by immunoblotting with appropriate antibodies.

Total RNA isolation and quantitative real-time polymerase chain reaction (RT-PCR)

Total RNA was extracted from cells by using the TransZol Up Plus RNA Kit (ER101-01, Transgen). An amount of RNA was reverse-transcribed to cDNA by using RevertAid RT Kit and random primers according to the manufacturer's instruction. Brilliant II SYBR Green qPCR Master Mix (Stratagene) and ABI 7900HT Real-Time PCR System (Life Technologies) were used. Target genes expression was normalized to the mRNA level of house-keeping genes. All the primer sequences are listed in Table S2.

siRNA and plasmid transfection

Cells were seeded into plates at 70% confluence. Cells were then transfected with gene-specific siRNA or control siRNA (10 nM) by using Lipofectamine RNAi MAX transfection reagent or plasmid with Lipofectamine 3000 (Thermo Fisher Scientific, Waltham, MA, USA) according to the manufacturer's instructions. Fresh medium was added at 6 h after transfection. Cells were cultured for another 48 h before treatment with the indicated reagents.

Flow cytometric analysis

Cells were fixed with 2% paraformaldehyde for 15 min and incubated with primary antibodies at 4°C for 30 min for flow cytometric analysis. The FITC-conjugated anti-SR-A1 antibody was from Abcam. For definition of positive and negative populations during the analysis, fluorescence minus one control was included in the experiments. The percentage of internalized SR-A1 was calculated as (SR-A1 at t = 0-SR-A1 at each time-point)/ (SR-A1 at t = 0) ×100%. Cell surface SR-A1 expression was determined using an FACSVerse flow cytometer (BD Biosciences) and the data were analyzed using FlowJo 7.6 software.

Lentiviral production

Vectors encoding Myc-SR-A1-WT-EGFP and Myc-SR-A1-K27R-EGFP carrying by pCDH-EF1-MCS-T2A-Puro (System Biosciences, USA), as well as two assistant vectors, pMD2.G, pSPAX2 were transfected into HEK293T cells. Viral supernatants were collected 48 h later, clarified by filtration and concentrated by ultracentrifugation.

Internalization assays

Cell surface proteins were labeled with EZ-link^{TM}-sulfo-NHS-SS-biotin (0.5 mg/ml) for 30 min at 4°C. To examine SR-A1 internalization, after biotinylation, ox-LDL was added into the culture media for different time-periods (0, 15, and 30 min). Residual biotin was removed from cell surface proteins using the reducing agent glutathione (50 mM) in a NaOH (100 mM), NaCl (75 mM) buffer (pH 8.6) for 30 min. A quenching buffer of 50 mM iodoacetamide in phosphate-buffered saline was added for 15 min before whole cell lysates were collected. Biotinylated proteins were pulled down at defined time points using avidin beads at 4°C. The beads were washed three times and analyzed by Western blot analysis. The average densitometric signal from western blot analysis of each assay at each time-point was used to

calculate the percentage of internalized SR-A1 as follows: (SR-A1 at each time-point - SR-A1 at t = 0)/ (SR-A1 immediately after cell surface biotinylation) ×100%.

Peptide synthesis and delivery

The peptides were synthesized by Scilight-Peptide Inc., Beijing, China, via Fmoc solid-phase peptide synthesis. The peptides were purified using a Varian ProStar 218 high-performance liquid chromatography (HPLC) system coupled to an Aglient Venusil MP reversed-phase C18 column. Peptides were eluted with a linear gradient of water, and acetonitrile (both containing 0.05% TFA) at a flow rate of 1 mL/min. The separation was monitored by UV detection at 220 nm. The peptides were then subjected to Voyager-DE STR mass spectrometric analysis. The solvents for gradient elution HPLC were as follows: solvent A, ACN 2%, TFA 0.05% and solvent B, ACN 90%, TFA 0.05%. The inhibitory peptides for blocking the interaction between USP9X and SR-A1 were designed based on the USP9X (M) binding region in SR-A1 consisting of amino acids (aa) 1–18. The 11 aa peptide (YGRKKRRQRRR) from the Tat protein transduction domain served as a cell-penetrating peptide. Thus, inhibitory peptides were chemically synthesized by linking with the N-terminus of the cell-penetrating peptide. FITC-labeled inhibitory peptides with FITC conjugated at the C- terminus were used to determine the presence of peptides in mouse lesions. The purity of synthesized peptides was verified by mass spectrometry and HPLC. For *in vitro* experiments, macrophages were treated with the peptide at 20 μM. For *in vivo* experiments, the indicated peptides (20 mg/kg) were injected intraperitoneally into mice three times per week for 10 weeks.

***In vitro* neutrophil Transwell migration and phagocytosis assays**

Bone marrow cells were harvested as described previously (2), and neutrophils were isolated

with the EasySep Mouse Neutrophil Enrichment Kit (STEMCELL Technologies) according to the manufacturer's instructions. For migration assays, neutrophil suspensions (1.0×10^6 cells) were added to upper chambers of transwell plates (BD Falcon, 353182) and the lower chambers were filled with medium alone or supplemented with CXCL1 (250-11, Peprotech). After incubation for 2 h at 37°C, the cells trapped in the membrane pores or adherent to the undersurface were fixed with 4 % paraformaldehyde, and stained with DAPI. Photographs of three random fields were taken and the cells were counted. For phagocytosis assays, neutrophils (1.0×10^6 cells) from *Usp9x*$^{flox/flox}$ or *Mac-Usp9x*^{KO} mice were seeded in black 96-well plates and were incubated with 100 µl of fluorescein-labeled *E. coli* BioParticles® (Vybrant™ Phagocytosis Assay, Molecular Probes, Invitrogen) suspended in Hanks' balanced salt solution. After incubation for 2 h, the suspension was then removed, and 100 µl of trypan blue suspension was added for 1 min to quench the extracellular probe. After aspiration of the trypan blue, the fluorescence in each well was measured on an EnSpire® Multimode Plate Reader at 484 (excitation) and 535 nm (emission).

Cholesterol efflux assay

BMDMs from *Usp9x*$^{flox/flox}$ or *Mac-Usp9x*^{KO} mice were treated with 50 µg/ml cholesterol for 24h. After washing, the cells were incubated with 50 µg/ml recombinant HDL (Yiyuan, Guangzhou, China) for 6 h as previously described (5). The cholesterol content in the medium and cell lysates was measured by mass spectrometry as previously described (6). The percentage cholesterol efflux was calculated as: (count in medium/total count) ×100%.

Mouse aortic macrophage isolation

Aortas removed from euthanized mice were cut into small pieces and digested into single cell

suspensions in an aorta dissociation enzyme stock solution (125 U/mL collagenase type-XI, 60 U/mL hyaluronidase type I-s, 60 U/mL DNase1, and 450 U/mL collagenase type-I; all enzymes were obtained from Sigma-Aldrich) at 37°C for 1 h and filtered through a 70-μm cell strainer as previously described (7). The cells were then resuspended and labeled with mouse anti-F4/80 magnetic microbeads (130-110-443, Miltenyi Biotec Inc.). Macrophages were collected by using MS columns (130-042-201, Miltenyi Biotec Inc.) on an OctoMACS™ Separator (130-042-109, Miltenyi Biotec Inc.).

Statistics

For all the experiments, effect sizes were estimated based on preliminary data, and selected cohort sizes for all experiments were sufficient to give a power of 0.8 at an α of 0.05. Analyses were conducted in a blinded fashion. No outliers were excluded. The number of replicates is indicated in the figure legends. The data were tested for normality before parametric statistical analysis using the Shapiro–Wilk normality test ($n < 10$). For normally distributed data, comparisons between two groups were performed using unpaired or paired two-tailed Student's *t*-test, and comparisons among multiple groups were performed using one-way or two-way ANOVA followed by a Bonferroni post-hoc test. Comparisons of non-normally distributed data were performed using the Mann–Whitney *U*-test. *P*-values are shown in the figures. Fisher's exact test was used to test the enrichment of differentially expressed proteins against all identified proteins in ubiquitylomics data. Adjusted P-values <0.05 were considered significant. Technical repeats, single patients, or mice are indicated by single symbols, if applicable. The statistical tests used for analysis of the data are stated in the figure legends for each experiment. GraphPad Prism (version 8.0) was used for statistical

analysis. All data are presented as mean ± standard error of the mean. $P < 0.05$ was set as the threshold for statistical significance.

Study approval

All operations towards human being were reviewed and approved by the Ethics Committee of Tianjin Medical University General Hospital (Tianjin, China), and written informed consent was obtained from all participants. All of animal experimental protocols were conducted in accordance with the National Institutes of Health Guide for the Care and Use of Laboratory Animals and approved by the Institutional Animal Care and Use Committee of Tianjin Medical University. (Tianjin, China).

Data Sharing Statement

For original data, please contact edin2000cn@163.com

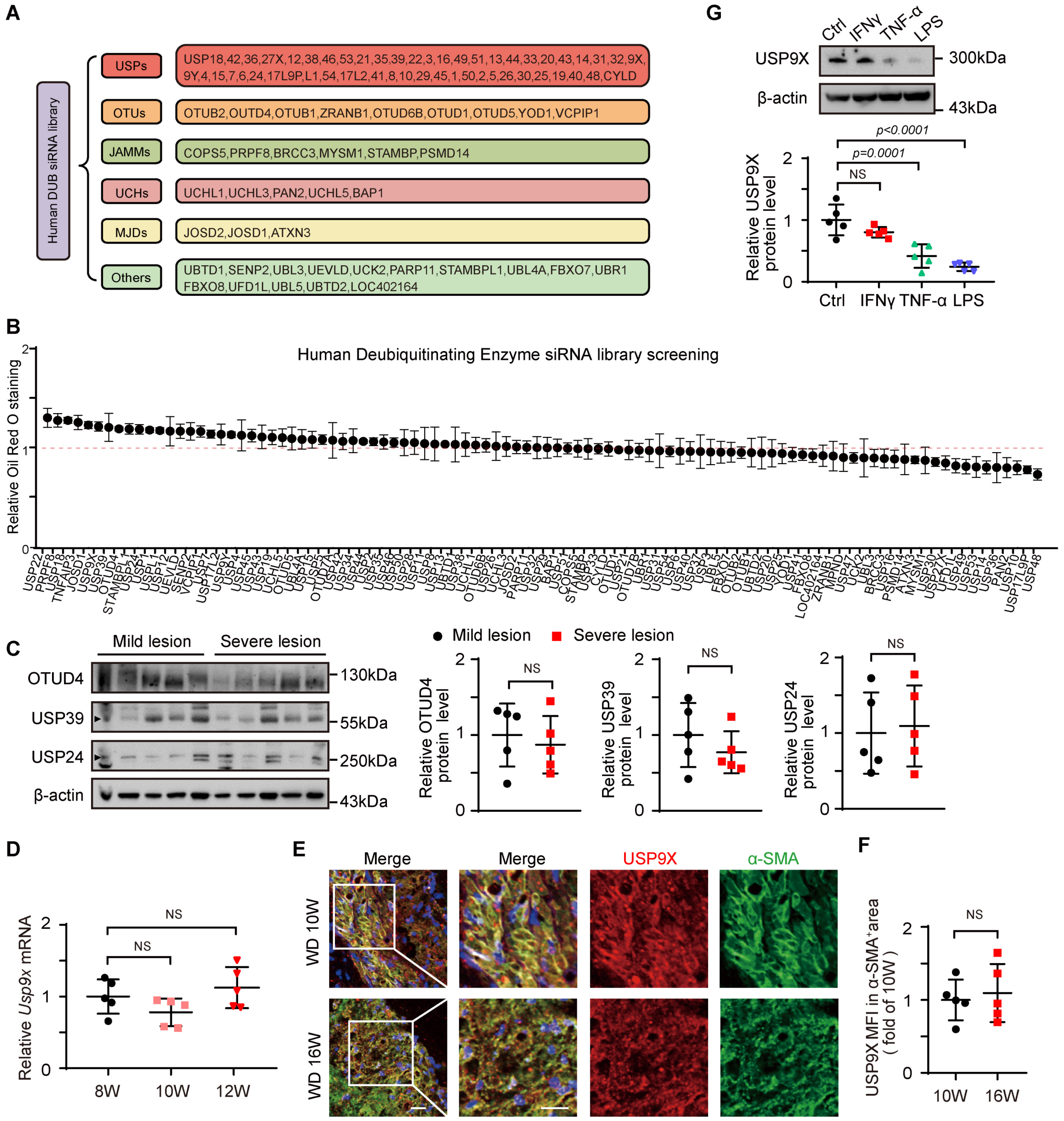

Supplemental Figures and Figure Legends

Figure S1. An *in vitro* screen identifies USP9X as potent suppressor of foam cell formation.

(A) Classification of human DUB siRNA library. **(B)** THP-1-derived macrophages were transfected with human DUBs siRNA for 48 h, and treated with ox-LDL (50 ug/ml) for another 24 h. Oil Red O staining was performed, and intensity of red coloration was quantified (n = 3). **(C)** Western blot analysis of the levels of OTUD4, USP39 and USP24

proteins in mild and severe atherosclerotic lesions in human carotid arteries. Paired two-tailed Student *t*-test (n = 5). NS, not significant. **(D)** Quantitative RT-PCR analysis of *Usp9x* mRNA levels in aortic macrophages from $Apoe^{-/-}$ mice fed a WD for 8, 10, and 12 wk. One-way ANOVA with Bonferroni multiple comparison post-hoc test (n = 5). **(E)** $Apoe^{-/-}$ mice were fed a WD for 10 and 16 wk. Immunofluorescence analysis of USP9X and α-SMA expression in aortic root sections. Scale bar, 50 μm. **(F)** Data represent the fluorescence intensity of USP9X in α-SMA^{+} areas (E). Unpaired two-tailed Student *t*-test (n = 5). **(G)** BMDMs were treated with TNF-α (50 ng/ml), LPS (300 ng/ml) or IFNγ (100 ng/ml) for 6 h. Western blot analysis of the protein levels of USP9X (left). Quantification of USP9X (right). Unpaired two-tailed Student's *t*-test (n = 5).

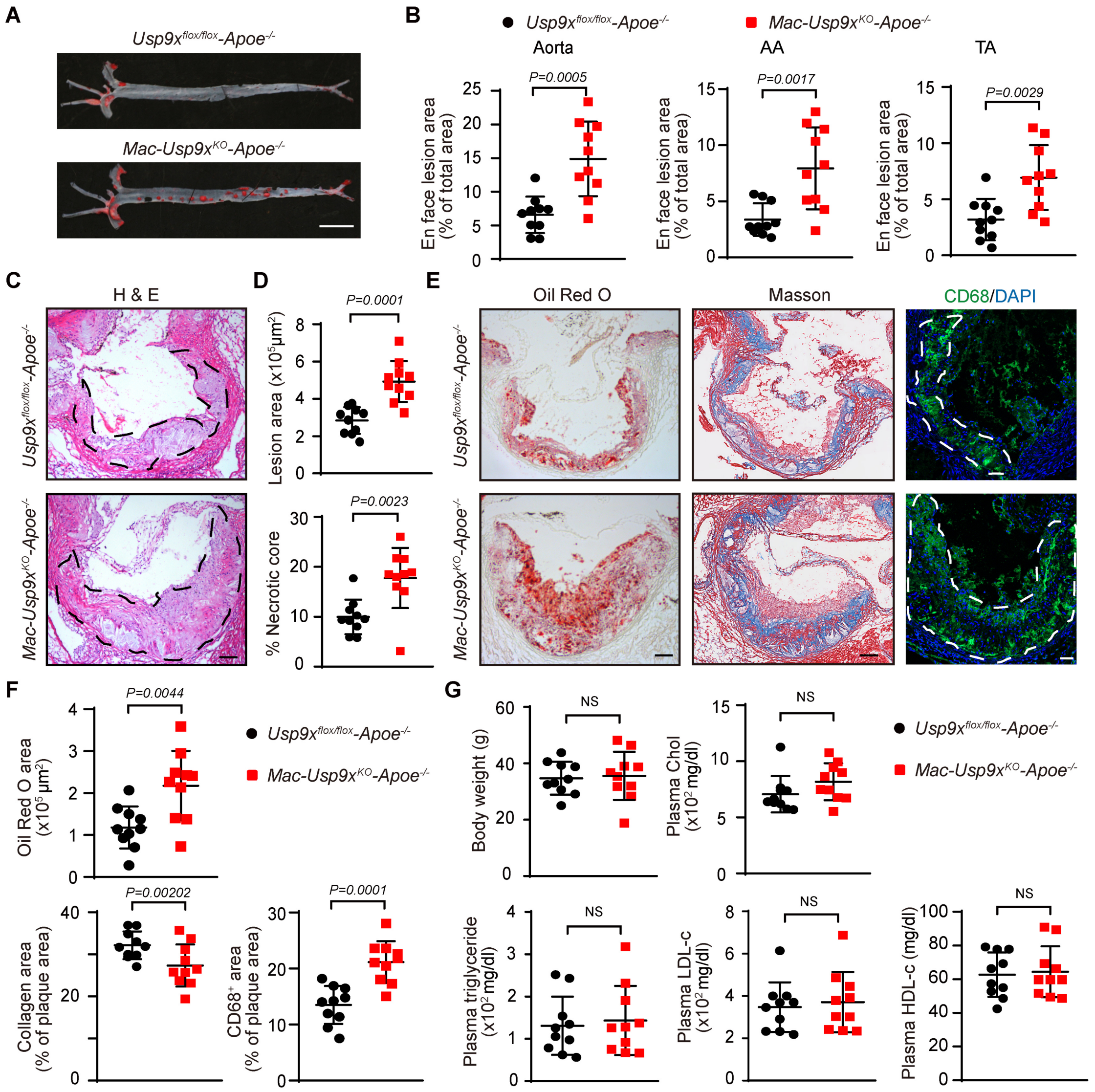

Figure S2. Macrophage USP9X deficiency promotes atherosclerosis.

(A) Oil Red O staining of aortas from male *Apoe*$^{-/-}$ and *Mac-Usp9x*^{KO}- *Apoe*$^{-/-}$ mice fed WD for 16 wk (n = 10). Scale bar, 5 mm. **(B)** Data represent the percentage of plaque area/total vessel area. AA, aortic arch; TA, thoracic aorta. Unpaired two-tailed Student's *t*-test (n = 10). **(C)** H & E staining of representative aortic root sections. Black dashed lines demarcate atherosclerotic plaques (n = 10). Scale bar, 100 μm. **(D)** Quantification of lesions area and percentage of necrotic core. Unpaired two-tailed Student's *t*-test (n = 10). **(E)** Oil Red O (left) and Masson's Trichrome (middel) staining of aortic root sections. Scale bar, 100 μm.

Macrophages identified by anti-CD68 antibody staining (right). The white dashed line indicates plaques. Scale bar, 50 μm. Unpaired two-tailed Student's *t*-test (n = 10). **(F)** Quantification of Oil Red O, collagen and CD68 positive areas in plaques. Unpaired two-tailed Student's *t*-test (n = 10). **(G)** Body and plasma levels of triglycerides (TG), cholesterol (CHO), low-density lipoprotein cholesterol (LDL-C) and high-density lipoprotein cholesterol (HDL-C). Unpaired two-tailed Student's *t*-test (n = 10).

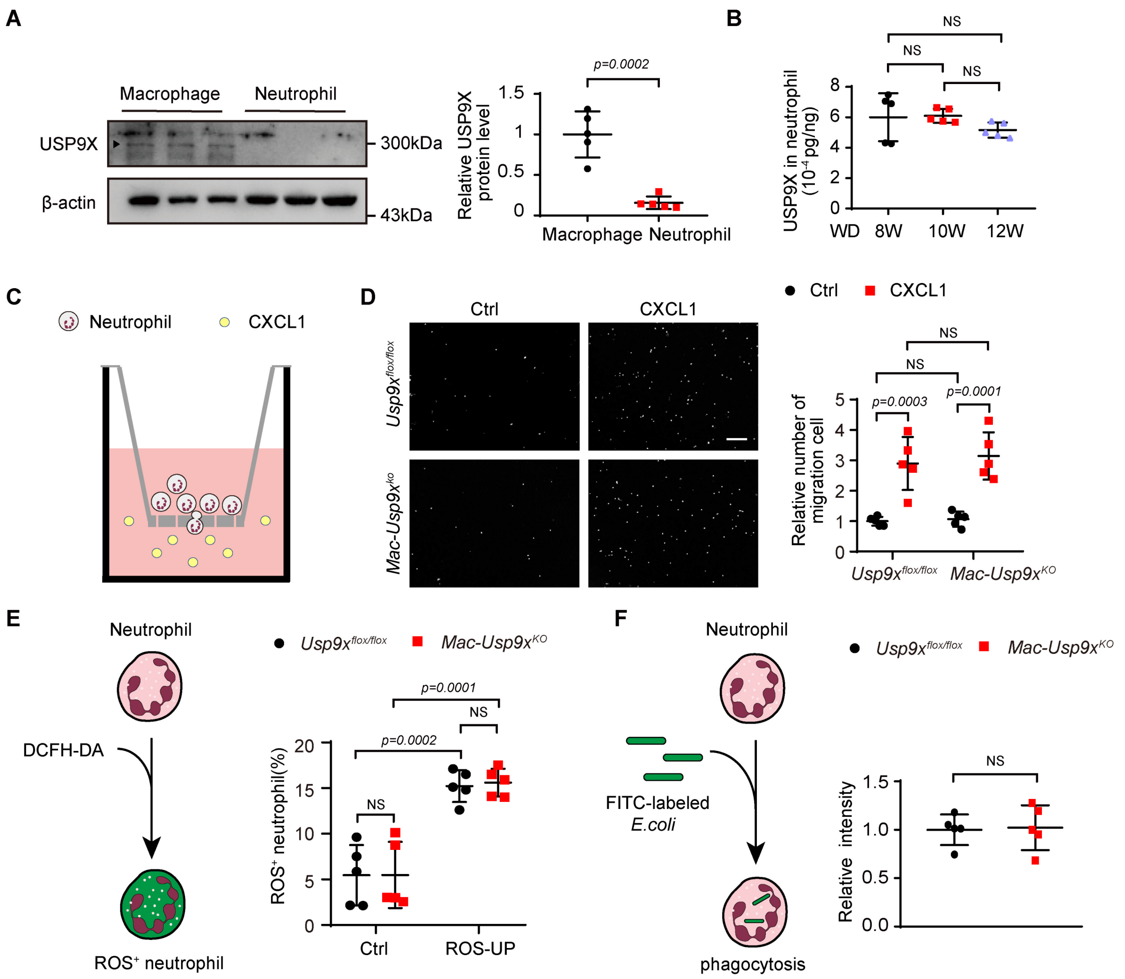

Figure S3. A lack of influence of USP9X in key functions of neutrophils.

(A) Western blot analysis of the protein level of USP9X in BMDMs and neutrophils from bone marrow of WT mice. Paired two-tailed Student t-test (n = 5). **(B)** ELISA assay of USP9X protein levels in neutrophils of aorta from *Apoe*$^{-/-}$ mice fed a WD for 8, 10, and 12 wk. One-way ANOVA with Bonferroni multiple comparison post-hoc test (n = 5). **(C-D)** Migration assay of neutrophils from *Usp9x*$^{flox/flox}$ and *Mac-Usp9x*^{KO} mice seeded into the upper chamber of transwell plates and incubated with medium containing PBS or CXCL1(100 ng/ml) for 2 h. Two-way ANOVA with Bonferroni post-hoc test (n = 5). Scale bar, 100 μm. **(E)** ROS production by neutrophils isolated from indicated mice as assessed by flow cytometric detection of DCF. Two-way ANOVA with Bonferroni post-hoc test (n = 5).

(F) Relative phagocytosis of FITC-labeled *E. coli* in neutrophils harvested from $Usp9x^{flox/flox}$ and $Mac\text{-}Usp9x^{KO}$ mice. Unpaired two-tailed Student's *t*-test (n = 5).

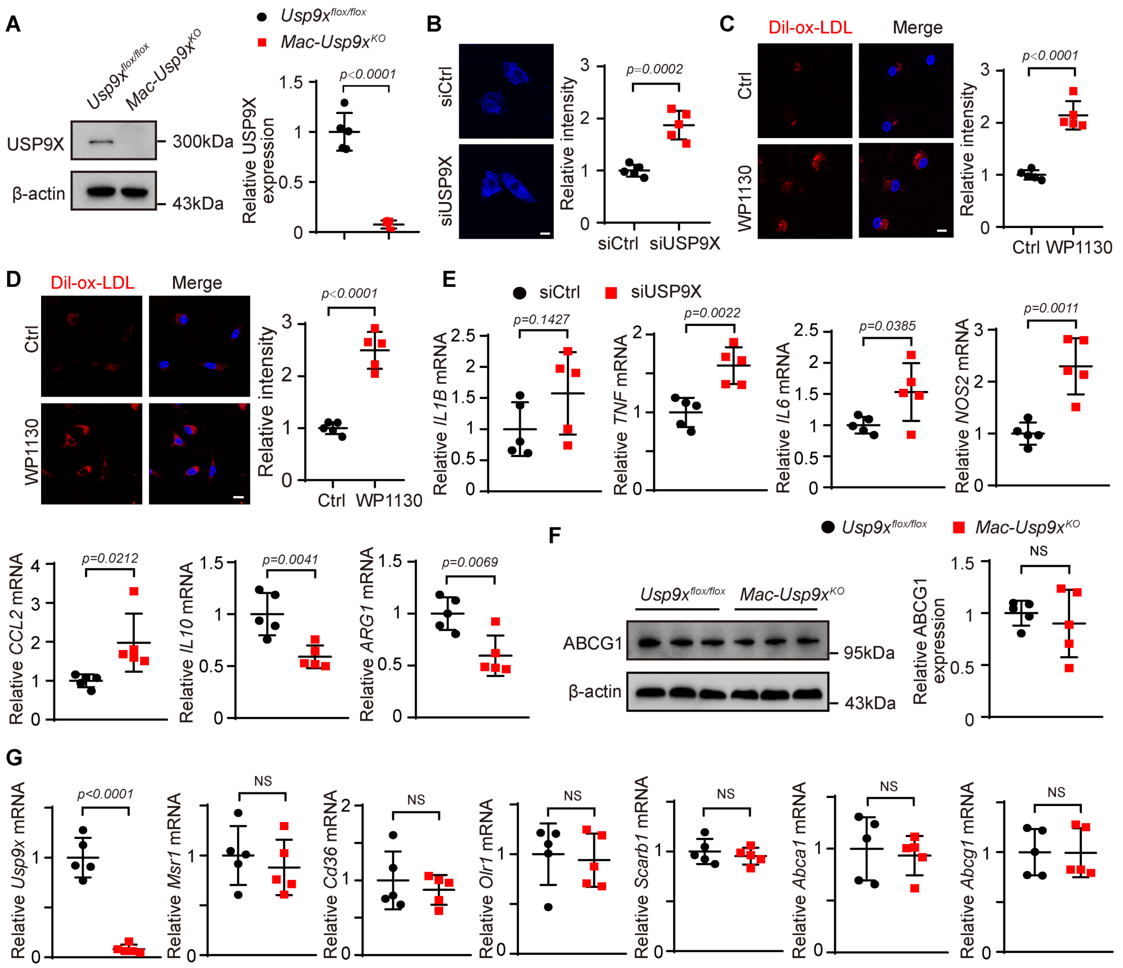

Figure S4. Macrophage USP9X deficiency increases lipid uptake and inflammatory genes expression.

(A) BMDMs were isolated from *Usp9x$^{flox/flox}$* or *Mac-Usp9x^{KO}* mice. Western blot analysis of USP9X was measured (left). Quantification of protein level of USP9X, which was normalized to β-actin (right). Unpaired two-tailed Student's *t*-test (n = 5). **(B)** HMDMs were transfected with siCtrl or si*USP9X* for 48 h, incubated with ox-LDL for another 24 h and stained with filipin. Scale bar, 10 μm. Unpaired two-tailed Student's *t*-test (n = 5). **(C)** Representative images of Dil-ox-LDL uptake by HMDMs pre-treated with or without WP1130 (5 μM) for 24 h. Scale bar, 10 μm. Unpaired two-tailed Student's *t*-test (n = 5). **(D)** Representative images of Dil-ox-LDL uptake by BMDMs pre-treated with or without WP1130 (5 μM) for 24 h. Scale

bar, 10 μm. Unpaired two-tailed Student's *t*-test (n = 5). **(E)** HMDMs were transfected with siCtrl or si*USP9X* for 48 h, and then treated with PBS or TNF-α (30 ng/ml) for 24 h. Quantitative PCR to detect the mRNA levels of the indicated genes. Target gene expression was normalized to the level of ACTB mRNA. Unpaired two-tailed Student's *t*-test (n = 5). **(F)** Western blot analysis of ABCG1 expression in BMDM from $Usp9x^{flox/flox}$ and $Mac\text{-}Usp9x^{KO}$ mice. Unpaired two-tailed Student's *t*-test (n = 5). **(G)** Quantitative RT-PCR analysis of indicated gene mRNA levels in BMDMs from $Usp9x^{flox/flox}$ and $Mac\text{-}Usp9x^{KO}$ mice. Target gene expression was normalized to the level of *Actb* mRNA. Unpaired two-tailed Student's *t*-test (n = 5).

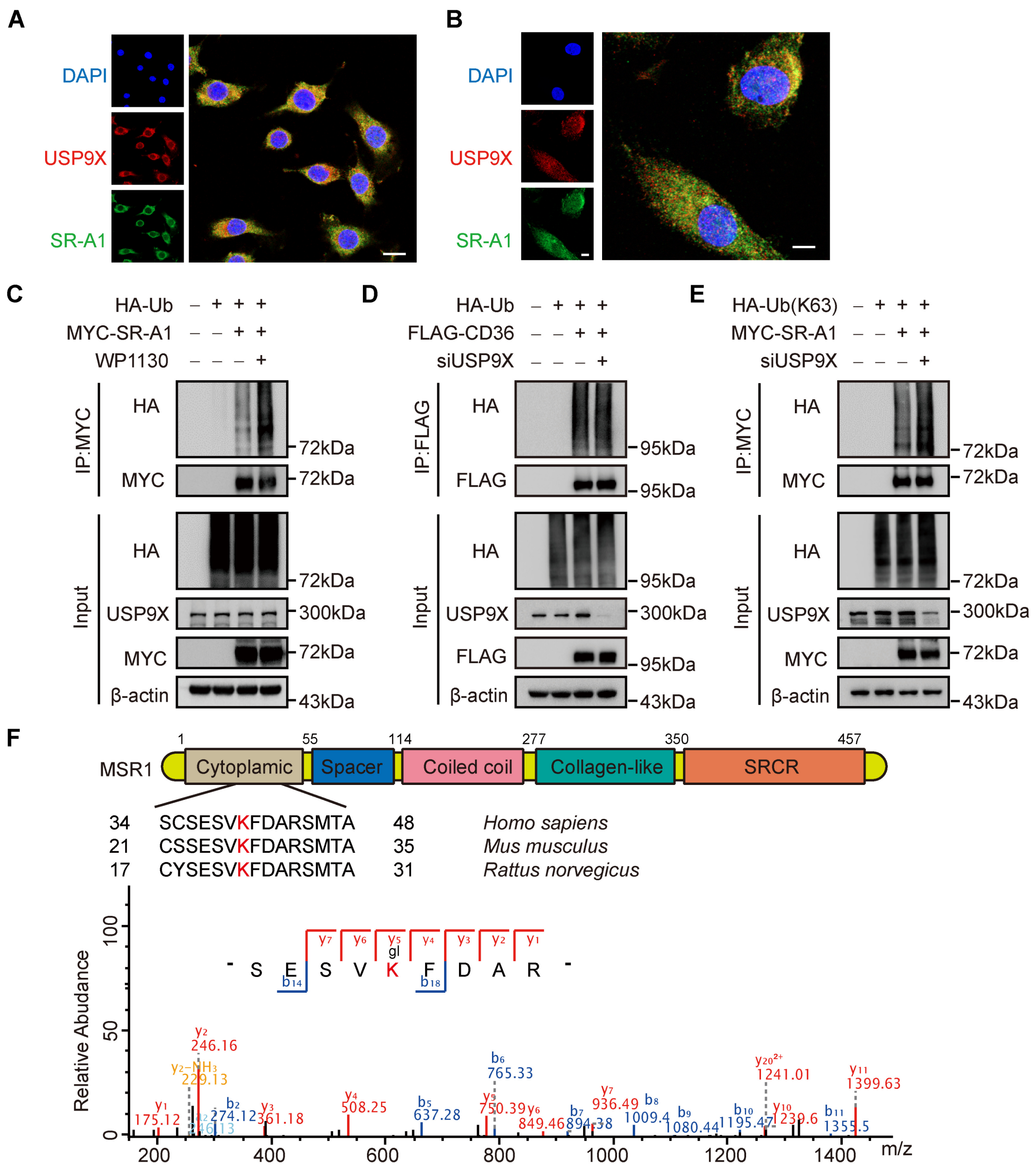

Figure S5. USP9X removes K63-linked polyubiquitination of SR-A1.

(A) Immunofluorescence staining of USP9X (red) and SR-A1 (green) in peritoneal macrophages (n = 5). Scale bar, 10 μm. **(B)** Immunofluorescence staining of USP9X (red) and SR-A1 (green) in HMDMs. Scale bar, 5 μm. **(C)** HEK293 cells were co-transfected with HA-Ub and MYC-SR-A1 for 48 h followed by treatment with WP1130 for 24 h and treated with MG132 for 2 h. The cells were subjected to IP with MYC magnetic beads followed by

immunoblotted with indicated antibodies (n = 5). **(D)** Western blot analysis of indicated proteins in HEK293 cells co-transfected with FLAG-labeled CD36 and HA-Ub in the presence of siCtrl or si*USP9X* plus MG132 for 2 h before IP of whole cell lysates with FLAG magnetic beads (n = 5). **(E)** Western blot analysis of indicated proteins in HEK293 cells co-transfected with MYC-labeled SR-A1 and HA-K63-Ub in the presence of siCtrl or si*USP9X* plus MG132 for 2 h before IP of whole cell lysates with MYC magnetic beads (n = 5). **(F)** Alignment of SR-A1 orthologs and secondary ion mass spectrometry analysis of SR-A1 ubiquitination modification sites.

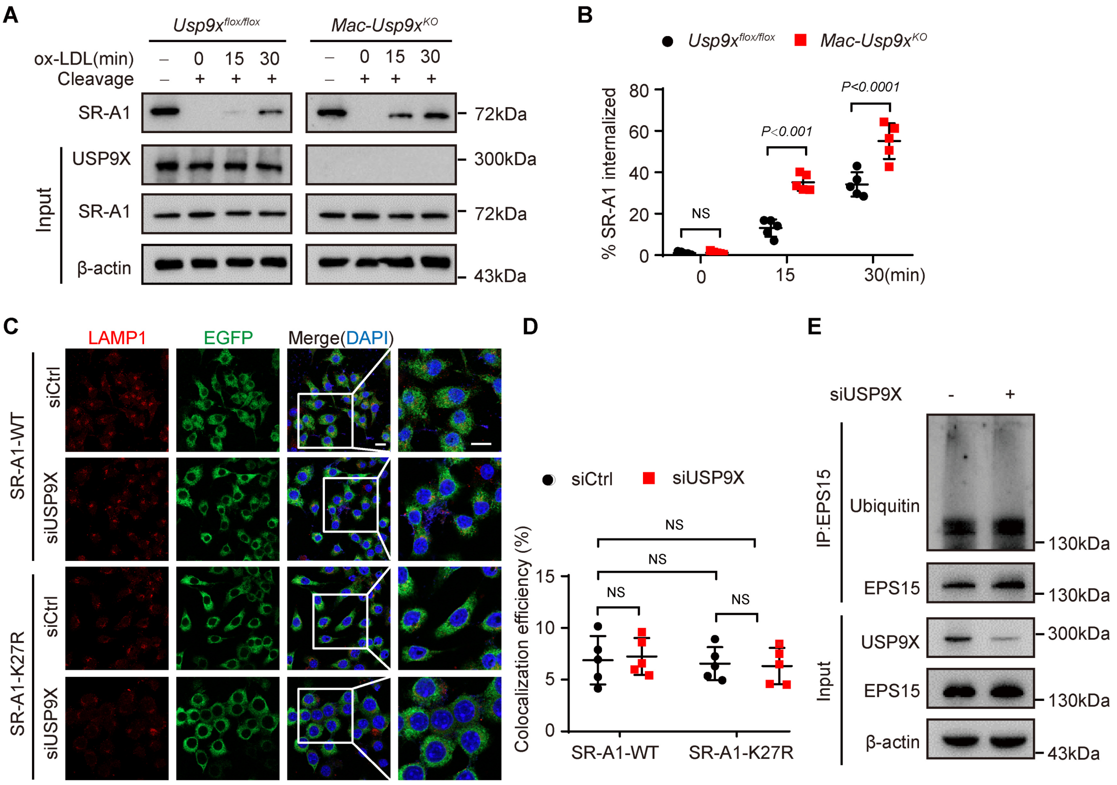

Figure S6. USP9X deficiency enhances ox-LDL-induced SR-A1 internalization in macrophages.

(A) BMDMs from *Usp9x*$^{flox/flox}$ and *Mac-Usp9x*^{KO} mice were subjected to surface biotinylation treatment that mentioned in supplemental methods, the internalized biotinylated proteins were pulled down followed by immunoblotting with indicated antibodies. **(B)** Quantification of SR-A1 internalization in (A). Two-way ANOVA with Bonferroni post-hoc test (n = 5). **(C)** Immunofluorescence analysis of RAW264.7 cells stably overexpressing SR-A1-WT-EGFP or SR-A1-K27R-EGFP and transfected with control or *Usp9x* siRNA for 48 h before incubation with ox-LDL for 1 h to induce internalization. Co-colocalization of SR-A1 (EGFP, green) and LAMP (red) is shown. **(D)** Quantification of the co-localization of the SR-A1 and LAMP1. Two-way ANOVA with Bonferroni post-hoc test (n = 5). Scale bar, 10 μm. **(E)** RAW264.7 cells were transfected with siCtrl or si*Usp9x* for 48 h and treated with MG132 for 2 h. The

cell lysates were subjected to IP with EPS15 antibodies followed by immunoblotting with the indicated antibodies (n = 5).

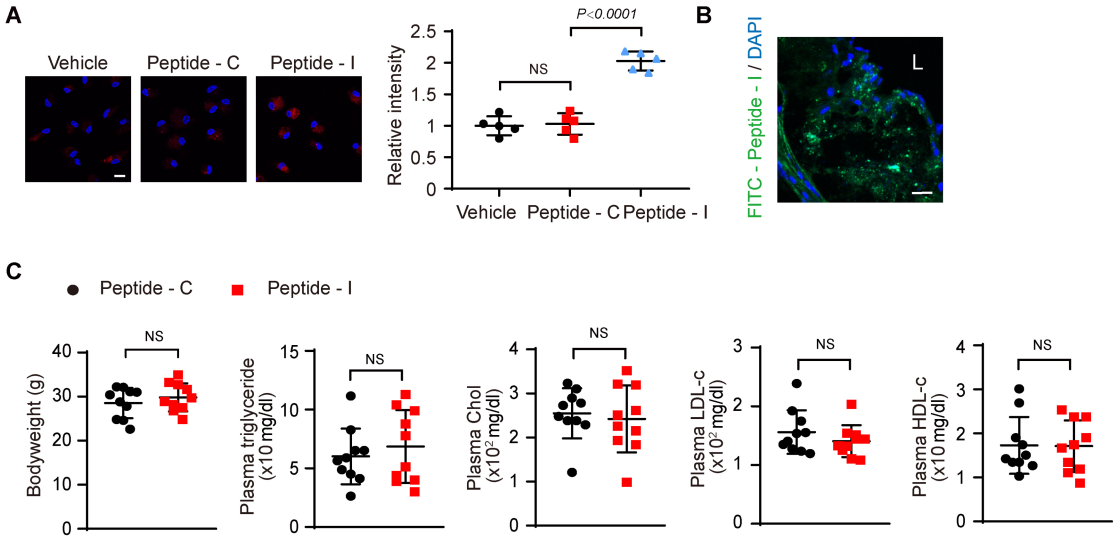

Figure S7. Blockade of the SR-A1–USP9X interaction using an inhibitory peptide promotes foam cell formation and atherosclerosis.

(A) Representative images of Dil-ox-LDL uptake by BMDMs pre-treated with or without the indicated peptides for 24 h. Scale bar, 10 μm. Unpaired two-tailed Student's *t*-test (n = 5). **(B)** *Apoe*$^{-/-}$ mice were intraperitoneally injected with FITC-labeled peptides and fed a WD for 10 wk. Representative image of aortic root section of mice injected with peptides. Scale bar, 20 μm. **(C)** Body weight and plasma levels of triglycerides (TG), cholesterol (CHO), low-density lipoprotein cholesterol (LDL-C) and high-density lipoprotein cholesterol (HDL-C). Unpaired two-tailed Student's *t*-test (n = 10).

Supplemental Tables

Table S1. Baseline characteristics of patients with atherosclerotic vessels

Patient ID	Age (years)	Sex	BG (mmol/L)	SBP (mmHg)	DBP (mmHg)	HDL (mmol/L)	LDL (mmol/L)	TG (mmol/L)	TC (mmol/L)
Patient 1	68	M	18	145	95	0.76	1.47	1.24	2.87
Patient 2	78	M	5.4	170	90	1.26	1.75	0.77	3.47
Patient 3	64	M	16	158	77	1.04	2.35	0.65	3.69
Patient 4	66	M	6.6	148	79	0.78	1.13	1.27	2.38
Patient 5	56	M	7.5	155	95	0.7	1.82	1.97	3.27

BG, blood glucose; SBP, systolic blood pressure; DBP, diastolic blood pressure; HDL, high-density lipoprotein; LDL, low-density lipoprotein; TG, triglycerides; TC, total cholesterol; M, male; F, female. n = 6.

Supplemental Table 2. List of gene-specific primer sequences used in this study

Target		Primer sequence
Actb (mouse)	Forward	GGCACCACACCTTCTACAATG
	Reverse	GGGGTGTTGAAGGTCTCAAAC
ACTB (human)	Forward	CATGTACGTTGCTATCCAGGC
	Reverse	CTCCTTAATGTCACGCACGAT
USP9X (human)	Forward	TTGCCTTGATTCCAACAGCC
	Reverse	AGTCGCCTGAGTGTTTAGCT
Usp9x (mouse)	Forward	GTAAACTGGGGCTTTGCCCA
	Reverse	TCAATGAAAAGAAACAAAGCCCAT
CCL2 (human)	Forward	CAGCCAGATGCAATCAATGCC
	Reverse	TGGAATCCTGAACCCACTTCT
TNF (human)	Forward	GAGGCCAAGCCCTGGTATG
	Reverse	CGGGCCGATTGATCTCAGC
IL-6 (human)	Forward	ACTCACCTCTTCAGAACGAATTG
	Reverse	CCATCTTTGGAAGGTTCAGGTTG
IL-1b (human)	Forward	ATGATGGCTTATTACAGTGGCAA
	Reverse	GTCGGAGATTCGTAGCTGGA
IL-10 (human)	Forward	TCAAGGCGCATGTGAACTCC
	Reverse	GATGTCAAACTCACTCATGGCT
ARG1 (human)	Forward	GTGGAAACTTGCATGGACAAC
	Reverse	AATCCTGGCACATCGGGAATC
NOS2 (human)	Forward	AGGGACAAGCCTACCCCTC
	Reverse	CTCATCTCCCGTCAGTTGGT
Il10 (mouse)	Forward	GCTCTTACTGACTGGCATGAG
	Reverse	CGCAGCTCTAGGAGCATGTG
Arg1 (mouse)	Forward	CTCCAAGCCAAAGTCCTTAGAG
	Reverse	AGGAGCTGTCATTAGGGACATC
Nos2 (mouse)	Forward	GTTCTCAGCCCAACAATACAAGA
	Reverse	GTGGACGGGTCGATGTCAC
Tnf (mouse)	Forward	ATGAGAAGTTCCCAAATGGC
	Reverse	CTCCACTTGGTGGTTTGCTA
Il1b (mouse)	Forward	GAAGAAGAGCCCATCCTCTG
	Reverse	TCATCTCGGAGCCTGTAGTG
Il6 (mouse)	Forward	AGTCCGGAGAGGAGACTTCA
	Reverse	TTCCACGATTTCCCAGAG
Ccl2 (mouse)	Forward	GAAGGAATGGGTCCAGACAT
	Reverse	ACGGGTCAACTTCACATTCA
Cd36 (mouse)	Forward	ATGGGCTGTGATCGGAACTG
	Reverse	TTTGCCACGTCATCTGGGTTT
Msr1 (mouse)	Forward	GGGAGACAGAGGGCTTAC
	Reverse	GGTTGATCCGCCTACAC
Scarb1 (mouse)	Forward	ATCTGGTGGACAAATGGAA
	Reverse	GAAGCGATACGTGGGAAT
Abca1 (mouse)	Forward	AGGGTGTCAGAGGTGTCTGTT
	Reverse	GGGTCGGGAGATGAGATGTG
Abcg1 (mouse)	Forward	CTTTCCTACTCTGTACCCGAGG
	Reverse	CGGGGCATTCCATTGATAAGG
Olr1 (mouse)	Forward	TTTCAGGTCCTTGTCC
	Reverse	CCATTCTCCCATAGCC

References

1. Alam S, Laughton DL, Walding A, and Wolstenholme AJ. Human peripheral blood mononuclear cells express GABAA receptor subunits. *Molecular immunology.* 2006;43(9):1432-42.
2. Zhang X, Goncalves R, and Mosser DM. The isolation and characterization of murine macrophages. *Current protocols in immunology.* 2008;Chapter 14:Unit 14 1.
3. Moore KJ, Andersson LP, Ingalls RR, Monks BG, Li R, Arnaout MA, et al. Divergent response to LPS and bacteria in CD14-deficient murine macrophages. *Journal of immunology (Baltimore, Md : 1950).* 2000;165(8):4272-80.
4. Li X, Song N, Liu L, Liu X, Ding X, Song X, et al. USP9X regulates centrosome duplication and promotes breast carcinogenesis. *Nature communications.* 2017;8:14866.
5. Shi Y, Lv X, Liu Y, Li B, Liu M, Yan M, et al. Elevating ATP-binding cassette transporter G1 improves re-endothelialization function of endothelial progenitor cells via Lyn/Akt/eNOS in diabetic mice. *FASEB journal : official publication of the Federation of American Societies for Experimental Biology.* 2018;32(12):6525-36.
6. Brown RJ, Shao F, Baldán A, Albert CJ, and Ford DA. Cholesterol efflux analyses using stable isotopes and mass spectrometry. *Analytical biochemistry.* 2013;433(1):56-64.
7. Butcher MJ, Herre M, Ley K, and Galkina E. Flow cytometry analysis of immune cells within murine aortas. *Journal of visualized experiments : JoVE.* 2011(53).
